# A Survey on Machine-Learning Techniques for UAV-Based Communications

**DOI:** 10.3390/s19235170

**Published:** 2019-11-26

**Authors:** Petros S. Bithas, Emmanouel T. Michailidis, Nikolaos Nomikos, Demosthenes Vouyioukas, Athanasios G. Kanatas

**Affiliations:** 1 General Department, National and Kapodistrian University of Athens, Thesi skliro, Psahna, 34400 Evia, Greece; 2 Department of Electrical and Electronics Engineering, Faculty of Engineering, University of West Attica, Ancient Olive Grove Campus, 12244 Aigaleo, Greece; emichail@uniwa.gr; 3 Department of Information and Communication Systems Engineering, School of Engineering, University of the Aegean, 83200 Samos, Greece; nnomikos@aegean.gr (N.N.); dvouyiou@aegean.gr (D.V.); 4 Department of Digital Systems, School of Information and Communication Technologies, University of Piraeus, 18534 Piraeus, Greece

**Keywords:** 5G networks, air-to-ground communications, machine-learning, unmanned aerial vehicles (UAVs), cellular networks

## Abstract

Unmanned aerial vehicles (UAVs) will be an integral part of the next generation wireless communication networks. Their adoption in various communication-based applications is expected to improve coverage and spectral efficiency, as compared to traditional ground-based solutions. However, this new degree of freedom that will be included in the network will also add new challenges. In this context, the machine-learning (ML) framework is expected to provide solutions for the various problems that have already been identified when UAVs are used for communication purposes. In this article, we provide a detailed survey of all relevant research works, in which ML techniques have been used on UAV-based communications for improving various design and functional aspects such as channel modeling, resource management, positioning, and security.

## 1. Introduction

Fifth-generation (5G) and beyond communications are mainly characterized by (i) massive connectivity, (ii) ultra-reliability and low latency, and (iii) increased throughput. Satisfying these objectives in conjunction with the rapid growth of the Internet of Things (IoT) applications represents a challenging task, especially in highly dynamic and heterogeneous environments. A promising approach is to adopt unmanned aerial vehicles (UAVs) as aerial user equipments (UEs) or flying base stations (BSs). In particular, UAV-based communications can improve the network performance in emergency situations by providing rapid service recovery and by offloading in extremely crowded scenarios. These characteristics have attracted the interest of the standardization bodies [1] and academia. Furthermore, the integration of artificial intelligence (AI) and machine-learning (ML) techniques in wireless networks can leverage intelligence for addressing various issues. Thus, the combination of AI/ML and UAVs appears to be strongly correlated in different disciplines and applications and throughout the network layers, promising unprecedented performance gains and complexity reduction. In the following subsections, a short introduction on the areas of UAVs and ML is presented while relevant surveys are discussed, identifying the current gap in the literature that has motivated the current work.

### 1.1. UAV Characteristics

As the demand for comprehensive broadband services, global coverage, and ubiquitous access has grown, non-terrestrial networks (NTNs) can strongly support the well-established terrestrial backhaul networks [2]. Among the principle components of NTNs, the low-altitude platforms (LAPs) intend to facilitate various civilian, commercial and governmental missions, as well as IoT applications [3], ranging from military and security operations to entertainment and telecommunications. UAVs, the major representative type of LAPs, is usually small unmanned aircrafts employed for short time periods (up to several hours) allowing the rapid deployment of a multi-hop communication backbone in challenging applications without any personnel involved, such as public safety, search and rescue missions, surveillance inspection, emergency communications in post-disaster situations or unexpected events, photographic reconnaissance, urban traffic surveillance, precision agriculture, and media traffic monitoring [4,5,6]. It is worth noting that the market research forecasts that the sales of UAVs will exceed $12 billion per year by 2021 [7], whereas the Federal Aviation Administration (FAA) predicts that the UAVs will count about 2.4 million units by 2022 [8]. It is obvious that such market size and dynamics have been the driving force towards the evolution of UAVs. Recently, great interest has risen by standardization bodies, industries, and academia towards the use of UAVs as flying BSs, mobile relays, or autonomous communicating nodes for providing low latency and highly reliable communications in cities, across suburban areas, and over rural terrains. The use of aerial vehicles for Long-Term Evolution (LTE) was suggested by the 3rd Generation Partnership Project (3GPP) [1], whereas the notion of nomadic relay was presented by the IEEE 802.16s Relay Task Group [9]. In 2013, realistic UAV frameworks have been also established by the special committee (SC-228) formed by the Radio Technical Commission for Aeronautics (RTCA) in an effort to frame performance specifications for the operation of UAVs [10]. Moreover, in 2016, RTCA constituted a drone advisory committee to safely introduce the UAVs into the national airspace system [11]. The National Aeronautics and Space Administration (NASA) and FAA have also launched a joint research initiative to integrate UAVs in national airspace system across the United States [12]. From an industry perspective, major vendors, i.e., Google, Facebook, Microsoft, Qualcomm, Nokia, and Huawei, have also tested the operability of aerial platforms for current LTE and future fifth-generation (5G) applications [13,14,15,16,17,18].

Depending on their flying mechanisms, UAVs can be classified into remotely piloted vehicles (RPVs), multi-rotor drones (also known as rotary-wings drones), fixed-wing drones, hybrid fixed/rotary wing drones, robot planes, and pilotless aircrafts and can differ in size from small toys to large military aircrafts [4,5,6]. Also, the payloads of UAVs including communication equipment, cameras, radars, and sensors vary from tens of grams up to hundreds of kilograms and directly determine their size, the battery capacity, and the flight duration. Owing to their unique characteristics, the UAVs are capable of providing ubiquitous and cost-effective wireless access over large coverage areas at high elevation angles and modest altitudes and with high chance of line-of-sight (LoS) connection with the ground nodes, while they ensure rapid deployment and movement on demand [4,5,6]. Apart from using a small number of UAVs, a swarm of UAVs can also cooperatively work to carry out complex tasks in significantly large areas [19] and especially in monitoring and surveillance applications, whereas the flying ad hoc networks (FANETs) [20], in which multiple UAVs communicate in an ad-hoc manner, can effectively expand the connectivity and communication range in scenarios with terrestrial network constraints, i.e., remote nodes, highly mobile nodes, and highly dispersed nodes. Nevertheless, challenges regarding the mobility, resource management, and control of the UAVs are imposed, especially due to large UAV swarms and the variability of their types, while the successful and long-term operation of UAV-based networks necessitates effective interference mitigation along with coordination and interoperability between heterogeneous wireless systems. Moreover, the limited endurance of the UAVs with respect not only to the networking and on-board processing tasks but also to the power demand of their engines and the flight control is currently one of the major practical factors restricting the full-scale deployment of UAVs in NTNs. In this respect, drone lifetime prolongation is a major concern, strongly related to flight characteristics and mission parameters.

Nevertheless, compared to terrestrial wireless networks, UAV networks have many distinctive features, such as highly dynamic network topologies, orbits or flight paths, and weakly connected communication nodes. Since the power supply is limited, energy-efficient design of airborne systems with respect to path planning and battery scheduling is also required to extend flight duration. Moreover, the mobility and the respective Doppler shift is increased and the Quality of Service (QoS) in data transmissions may be asymmetric. Overall, the communication requirements need to be adapted to the rate and quality of the data transmission in order to achieve the desired performance metrics. Additionally, existing conventional communications techniques encompass inherent limitations, particularly in the case of complex communication scenarios, where unexpected and nonlinear phenomena prevail. As dynamic and mission-critical UAV-based communications lead to particular complexity, uncertainty, and high degree of variability, AI/ML is the key technology that enables fundamentally different decision-making capabilities to obtain a proper UAV placement and trajectory.

### 1.2. Artificial Intelligence and Machine Learning

AI has been considered the science of training machines in order to perform human tasks. There are many applications that AI has been involved in, including robotic vehicles, speech recognition, machine translation, and recently wireless communications. Moreover, a specific subset of the AI is the techniques that are used for training machines in how to learn, which originates a new framework known as ML. In this context, ML can provide solutions in scenarios where a massive number of devices simultaneously requires access to the network’s resources in a dynamic, heterogeneous, and unpredictable way, e.g., in IoT communications. In this sense, intelligent management should be performed in the entire network in order to cope with the various demanding requirements of this novel type of services. The scope is to adaptively and in real-time manage the network resources in an optimal manner. Therefore, ML algorithms have been proposed as an efficient approach for confronting all these contradictory challenges coming from the IoT ecosystem.

In general, ML is based on the pattern recognition framework and its main idea is to exploit correlation among a set of data and/or past good action sequences for adapting to the environmental changes without any kind of human intervention. Clearly, the advantage offered by the ML framework in the wireless networks operation is that it will enable network elements to monitor, learn, and predict various communication-related parameters, such as wireless channel behavior, traffic patterns, user context, and devices locations. ML is classified into various categories, such as supervised learning, semi-supervised learning, unsupervised learning, and reinforcement learning (RL) [21].

- **Supervised learning:** In supervised learning, the algorithms use data sets, in which both the input and the desired output are available. Therefore, this kind of algorithm can only be employed in scenarios where enough labeled data are available to be exploited.
- **Unsupervised learning:** The unsupervised learning algorithms also require data to be available for training, which, however, do not include labeled output. Therefore, in this type of learning, clustering or pattern discovery is performed on the available data.
- **Semi-supervised learning:** An intermediate approach regarding the nature of the available data has been followed with the semi-supervised learning algorithm. In this type of learning, both labeled and unlabeled data are exploited for the training.
- **Reinforcement learning:** In RL, the problems are solved by employing a sequence of actions that use the trial and error rule. Therefore, the main idea of this type of learning is radically different as compared to the previous mentioned ones, which exploit historical data. Instead, RL algorithms are trained by the previous taken decisions towards solving the problem. The RL algorithms are used in various scenarios in the area of wireless network optimization.

In addition, a specific class of ML is deep learning (DL). In DL, multiple layers have been employed in order to build an artificial neural network, which is able to make intelligent decisions without any kind of human intervention. DL algorithms can be applied when limited manual interference is necessitated, with the cost of higher computational requirements. Nevertheless, AI, ML, and DL methods have been widely employed in various wireless communications scenarios for improving a numerous parameters of the network.

### 1.3. Previous Survey Works

Along with the growing number of novel solutions for wireless networks during the last years, several recent surveys focusing on the interplay of AI/ML and wireless communications have been provided. Various studies have proposed the application of ML for improving the performance of wireless networks. The authors in Reference [22] provide a brief description on the use of DL in an architecture based on the aerostack framework, which is an aerial robotics architecture consistent with the common components related to perception, guidance, navigation, and control of unmanned rotorcraft systems, with different robotic agents consisting of different abstraction levels of unmanned aerial robotic systems, like social, reflective, and deliberative layer, among others. The DL algorithms are employed in three directions: feature extraction, planning and situational awareness, and motion control. The classification is limited to the learning type of the algorithm and the field of application for aerial robot systems which use specific sensors. Next, the survey in Reference [23] studied the use of deep reinforcement learning (DRL) for several network and communication aspects, such as network access and rate control, cashing and offloading, security and connectivity preservation, routing, resources, and data collection. In some cases, the UAV is considered as an agent for the cellular network, like a base station, a sensor, a mobile, a user, a network controller, or a scheduler. Deep Q-network (DQN), deep Q-learning (DQL), and liquid state machine (LSM) are some of the learning algorithms used for the aforementioned issues for the UAV scenarios. A rather comprehensive survey from the exclusive angle of DL applications in mobile and wireless network is depicted in Reference [24]. The authors provide classifications for different attributes, such as mobile big data, data and mobility analysis, user localization, network control, and security. However, the use of UAV networks is not taken into consideration. At the same time, several of the DL techniques that are discussed can be of importance to UAV networks. Then, the survey in Reference [25] provides a brief overview of ML techniques for wireless big data analytics. One of the described applications is related to UAVs, still without discussing AI/ML aspects. Another work discusses, among others, that the AI/ML algorithms on edge devices in 5G and beyond 5G networks are necessary for the development of 6G networks [26]. The critical applications of self-sustaining networks (SSNs) in 6G requires low-latency, high-reliability, and scalable AI, along with a reliable infrastructure, relying on the integration of UAVs and ground network nodes.

In networks consisting of ground-based and aerial vehicles, there have been various surveys presenting possible applications of AI/ML. First, the exploitation of AI for vehicle-to-everything (V2X) application is given in Reference [27], including comparisons of the AI algorithms. Moreover, in autonomous driving, the survey categorizes the most widely used AI techniques in swarm intelligence, ML, DL, expert systems and planning, scheduling, and optimization, where one may find similarities to the prominent UAV classification. Some of the suggested open source or proprietary software tools can also be used in UAV communications, but most of them consider applications like safety, network congestion, navigation, security, content delivery, and edge computing. Furthermore, the paper in Reference [28] gives an overview of 5G communication aspects for V2X, UAVs, and healthcare use-cases. More specifically, UAV-based wireless networks aspects are mostly discussed for disaster management purposes without considering ML enhancements, while ML is mainly considered for healthcare applications in the area of anomaly detection for patients. Next, the authors in Reference [4] present the integration of UAV networks for 5G and beyond communications. Several newly founded techniques are considered (e.g., non-orthogonal multiple access (NOMA) and millimeter wave band (mmWave) transmissions), while a classification of different techniques is performed in terms of physical layer; network layer; and joint communication, computing, and caching, taking into consideration the BS type and the number of UAVs. However, only a limited number of works on the application of ML in UAV networks is included. Then, the survey in Reference [6] offers a thorough overview on using UAVs for cellular communications in view of experimental and simulation channel studies that have been performed in such networks, along with prototyping. In the latter, only two platforms are presented making use of ML algorithms for trajectory and placement. The rest of the paper is dedicated to standardization, regulation, and security aspects, while ML is only included as a possible future research direction for UAV networks.

From the cyber-security point of view, one may find a limited number of survey papers, studying AI/ML. The article in Reference [29] provides a well-structured review of UAV networks from a cyber-physical system (CPS) perspective. The three CPS components in the UAV networks that the survey analyzes are communication, computation, and control. Regarding AI/ML techniques, the authors classify open-source software projects and libraries, mainly focusing on the machine vision area. The authors briefly mention the use of ML and RL at the physical layer and routing, correspondingly, at the computation and communication components. Regarding the computation and control components, intelligent algorithms are discussed for computation-enhanced flight and formation control. The survey in Reference [30] does not focus on communication issues, while the authors provide a mapping of UAV network connectivity, QoS, security, design challenges, and requirements for cyber-physical (CP) security applications. The application of ML is presented for object detection and image recognition, as well as for interesting future research areas, such as collision avoidance, spectrum sensing, channel estimation, and energy management. The main goal of Reference [31] is to outline the wireless and security challenges that arise in the context of UAV-based delivery systems, real-time multimedia streaming, and intelligent transportation systems. To address such challenges, artificial neural network (ANN)-based solution schemes are introduced. The authors consider the security at higher communication layers. Regarding the wireless challenges and relevant AI/ML solutions, each use case follows a different approach and ANN-based solution. Also, the authors provide a discussion on physical layer communication issues, such as interference management. Finally, the work in Reference [5] provides a comprehensive study on the use of UAVs in wireless communications and networks from the CP security perspective. Two main use cases of UAVs are investigated: aerial wireless BSs for 5G and beyond applications that complement emerging wireless communication systems and cellular-connected users that use existing wireless infrastructure. For each use case, key challenges, applications, and fundamental open problems are noted, along with the mathematical tools and techniques needed for addressing the challenges. Regarding the latter, the tutorial provides only a small section discussing the emerging field of ML, primarily for trajectory and path planning.

The majority of surveys either focus on ML for different wireless network environments or foresee the integration in UAV-based networks, discussing its future potential. Nonetheless, three recent works can been identified as more similar to this study. First, the survey in Reference [32] focuses on AI for robotic applications, comprising both ground-based nodes and UAVs. In such scenarios, UAVs can improve the connectivity and security of multi-robot teams towards efficiently performing their tasks. AI/ML techniques for aerospace robotics include (i) ANN for delay, connectivity and security improvement, as well as channel prediction; (ii) particle swarm optimization for determining UAV trajectory; (iii) DL for improved UAV connectivity; and iv) ML for user content request prediction from the UAVs. Then, the article in Reference [33] focuses on a multi-layered communication system comprising space (satellite), air (UAV), and ground segments, namely space-air-ground integrated network (SAGIN). It is found that DL, mainly convolutional neural networks (CNNs), with different DL architectures and training methods can improve the network performance. Next, the tutorial in Reference [34] provides a classification of ANNs for wireless communications. Among several applications (e.g., caching, multiple radio access technologies (RATs), and IoT), the authors refer to UAVs and discuss RL for coverage, connectivity, trajectory, resource, and path planning optimization. A cellular use case for applying AI is considered, where UAVs are part of cache-enabled BSs. Finally, recurrent neural network (RNN) and especially echo state network (ESN) algorithms at the conceptor are studied for mobility prediction.

Departing from these works, in this survey, we focus on the application of a broad set of AI/ML techniques for UAV-based communications without focusing on particular AI/ML categories or UAV applications. More specifically, the following contributions are given:

- An exhaustive overview of AI/ML solutions from all possible categories and their application in UAV-based networks is presented.
- A wide range of UAV-enhanced wireless communication issues, ranging from physical layer and resource management aspects up to trajectory design and caching is studied, while wireless security and public safety applications are comprehensively discussed.
- Open issues are identified for both networking and security areas, stimulating further research for the application of AI/ML techniques in UAVs-based networks.

Table 1 provides brief descriptions of the relevant surveys and the research areas and scope in the context of AI/ML that they discuss.

**Figure 1:**
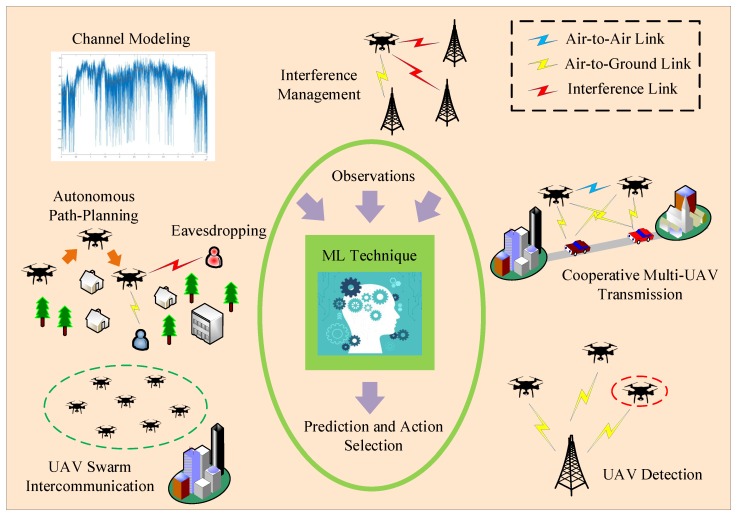
Applications of the AI/ML in UAV-based communications.

Also, in Figure 1, various applications of the AI/ML solutions in UAV-enabled communications are depicted. Generally, the importance of distilling intelligence in wireless communication networks has been outlined in numerous works. In Reference [24], the authors have observed that the ever increasing heterogeneity and complexity of mobile networks has made monitoring and management of network elements intractable. Moreover, ML allows systematic mining of valuable information from mobile data and automatically identifies correlations that are too complex to be derived by human experts. Likewise, the work in Reference [33] has noted that, in wireless networks, ML enables the wireless devices to actively and intelligently monitor their environment, exploiting mobile data for training purposes in order to learn, predict, and adapt to the evolution of environmental features, including wireless channel dynamics, traffic and mobility patterns, as well as network composition, among others. In this way, they can proactively act towards maximizing the probability of satisfying different performance metrics. It can be seen that ML takes as input data from different sources and through the application of various learning techniques, i.e., supervised/unsupervised, deep or reinforcement learning, allows the network to adapt to the wireless environment in a dynamic and autonomous manner. Thus, especially in networks consisting of a large number of nodes, such as those consisting of swarms of UAVs [35], centralized coordination and excessive overheads that must be acquired and exchanged among the network nodes are avoided, paving the way for distributed network optimization where intelligence plays a key role.

**Figure 2:**
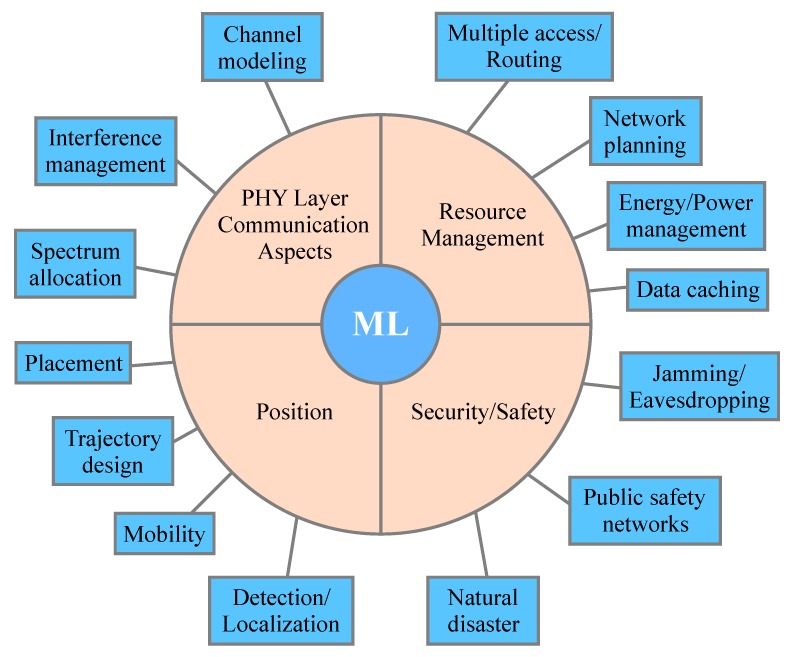
Classification of the AI/ML solutions in UAV-based communications.

Moreover, Figure 2 provides a classification of UAV-based communication network aspects, where the adoption of ML techniques can enhance their performance compared to conventional optimization techniques. Stemming from this taxonomy, the rest of the paper is organized as follows. In Section 2, we survey communication aspects related to the physical layer, such as channel modeling and interference management. In Section 3, we survey security and safety issues, while in Section 4, we present ML-based researches related to resource managements and, in Section 5, we survey applications related to UAVs positions. Finally, Section 6 presents open issues and research challenges in the context of ML applications in UAV-enabled communications and Section 7 concludes this article.

## 2. Physical Layer Issues

A wide range of communication aspects exists that can be enhanced through ML solutions, including (i) the development of accurate channel models in various environments and the mitigation of path-loss (PL) through prediction of the topology; (ii) the tackling of severe interference from other UAVs and from ground nodes with timely training, using mobility and user association data; and (iii) the configuration of transmission parameters towards achieving specific performance targets.

### 2.1. Channel Modeling

The accurate prediction of channel behaviour in wireless networks is of utmost importance, especially in highly mobile environments. The selection of appropriate channel models is necessary for performance evaluation, network planning, and UAV trajectory design among others. Towards this end, various ML solutions have been proposed for predicting the channel behaviour and UAV received signal strength (RSS). ML techniques are capable of assessing the wireless conditions without requiring a vast amount of data sets, as it is the case in channel modeling from extensive measurement campaigns. Motivated by the scarcity of UAV measurements in real environments, the authors of References [36,37] use ANNs and ensemble methods for predicting the RSS at the UAVs. First, in Reference [36], a multiple-layer perceptron (MLP) NN is used and trained by measurement data. Novel training methods are developed based on the combination of self-adaptive differential evolution (DE) algorithms with the Levenberg–Marquardt (LM) method. The sequential application of DE and then LM method offers better initial starting weights compared to the case of random weight generation, resulting in better solutions and faster convergence. Therefore, various new algorithms for ANN training can be devised employing DE in the exploration phase and LM in the exploitation phase. The authors propose to use the success-history-based adaptive DE with linear population size reduction (L-SHADE) algorithm for ANN training. L-SHADE self-adapts the control parameters and performs linear population size reduction. Performance comparisons with four DE algorithms showed that L-SHADE provides the best performance due to its unique features, being a promising solution to extract channel model characteristics without relying on large amounts of measurement data. The study in Reference [37] focused on ensemble methods for UAV RSS prediction. The operation of ensemble methods was based on training combinations of supervised learning base learners, i.e., methods such as decision trees or NNs, among others. This work extended the NN-based algorithm of Reference [36], developing a new ensemble method relying on five base learners, namely support vector machines (SVMs), GP, ANNs, least squares boosting method (LSBoost), and bagging prediction. At the same time, the ML-based solution adopts the salp swarm algorithm (SSA) evolutionary algorithm, inspired by the swarm movement and foraging of salps in the oceans. In greater detail, the behavior of salps is modeled by dividing them in two groups, consisting of leaders and followers. The first group includes salps with higher objective function values, while the second group contains salps with lower objective function values. The proposed models are validated by appropriate performance indicators, i.e., the mean absolute error (MAE), the root mean squared error (RMSE), and the mean absolute percent error (MAPE). It was shown that, by using the ensemble method, the accuracy of channel modeling can be improved when compared to NN-based approaches, proving that ensemble learning can be a valuable tool to solve problems in the field of electromagnetics.

In another line of research, the study in Reference [38] examines real-time three-dimensional (3-D) wireless channel modeling of the air-to-ground channel among UAVs and ground nodes, using unsupervised learning. In greater detail, a clustering technique is employed to analyze the 3-D wireless channel LoS and Non-Line-of-Sight (NLoS) channel states based on RSS measurements by the UAV. Additionally, PL and shadowing parameters are derived, formulating an appropriate temporary 3-D channel model. As an online learning approach is followed, changes in the communication environment are integrated in the temporary 3-D channel, increasing its accuracy. As shown in the performance evaluation, employing an online learning method allows temporary 3-D channel modeling to be derived according to dynamic changes of the wireless environment. As a result, channel modeling accuracy can be significantly increased in UAV communication scenarios. Nonetheless, the impact of shadowing is not considered in this work, limiting the accuracy of the 3-D channel modeling.

In Reference [39], a method that optimizes the positioning of UAVs acting as aerial relays in air-to-ground wireless networks was presented. This method enables the autonomous path planning of UAVs by exploiting a finely structured radio map. Since this map cannot be easily obtained in practice due to the complex terrain in common urban propagation areas, a joint clustering and regression problem using a maximum likelihood approach was formulated based on the K-segment ray tracing model. In particular, an ML-based iterative algorithm was employed, acting as a channel predictor in an effort to reconstruct the radio map from a limited number of training samples via a kernel-based method. The results pointed out that the proposed method requires almost as few as one-tenth of training samples to achieve the reconstruction performance of the K nearest neighbor (KNN) baseline method, while it can recognize the propagation segments and, hence, optimizes the placement of the UAVs.

Furthermore, the accurate modeling of PL prior to the installation and operation of a wireless network is a complex process requiring data from a detailed link budget analysis. Currently, radio engineers mainly adopt deterministic and stochastic methods for estimating signal attenuation due to PL. It must be noted that, in irregular terrains characterized by vegetation, hills, and buildings, PL models provide limited accuracy of signal attenuation. In order to separate the impact of these factors on the PL, the work in Reference [40] relied on geo-referenced satellite imaging and 3-D point cloud in order to extract the environmental characteristics through 3-D image classification and to feed them in ML-based PL modeling for improved wireless network deployments. Therefore, light detection and ranging (LiDAR) and ANNs are employed, resulting in a simulation environment identifying features from the environment, such as tree foliage. Then, these features are used to adjust the RSS calculation through the PL model. Performance evaluation was based on MAPE, revealing that the proposed PL prediction method significantly outperformed existing empirical PL models, such as COST 231-Hata and log-normal, due to the consideration of environmental irregularities. In greater detail, empirical PL models exhibited RSS estimation errors within 6.29–16.9% from the actual RSS measurements while the proposed ML-based solution significantly reduced the estimation error to 4.26%. Still, the effect of buildings was not considered, limiting the applicability of this method in urban settings.

Aiming to study the application of ML on PL prediction for the air-to-air channels, the authors of Reference [41] proposed various ML-based PL models for urban environments. The considered ML algorithms consisted of the random forest (RandF) and KNN. RandF is an ensemble method stemming from decision trees. In order to perform classification, it collects classifications from all the decision trees and selects the most popular prediction as its output, forming a strong classifier from a large number of weak or weakly correlated classifiers. KNN performs supervised learning based on a similarity measure, calculating the distance between points on a graph in order to solve classification or regression problems. Then, using ray-tracing to generate training and testing data, the prediction accuracy of the ML-based PL models was compared with the empirical Stanford University Interim (SUI) and the COST231–Walfisch–Ikegami models. It was shown that the ML-based models achieved better air-to-air PL prediction by relying on training data related to propagation distance, UAV altitude, elevation angle, and LoS conditions. Moreover, among the ML-based approaches, RandF offered the best prediction results while the most important data was that of path visibility with propagation distance and elevation angle, also having significant influence. Another study focused on PL prediction for UAV networks communicating with ground nodes in Reference [42]. However, departing from radio-frequency (RF) PL prediction, the authors examined instead, PL and delay spread prediction in mmWave channels, employing the RandF and KNN ML algorithms. Moreover, the problem of requiring massive data sets for training of the ML algorithms is mitigated through transfer learning based on limited data sets and training results that were acquired from previous training phases. From the results, it is concluded that the proposed methods offer reduced mean square errors when compared to Okumura–Hata and COST 231–Hata models.

### 2.2. Interference Management

A major issue arising not only in UAV networks but also in wireless networks in general is interference. Recently, ML-based approaches have been invoked for efficient interference management in UAV networks. The paper in Reference [43] studied opportunistic channel access by UAVs. This problem was formulated as a noncooperative interference mitigation game, considering the distinct characteristics of data traffic and UAV clustering. After, these characteristics are inserted in the utility function, appropriately allocating the weight coefficients to each characteristic and linearly combining them are performed. Moreover, a distributed log-linear learning algorithm is employed to achieve the Nash Equilibrium (NE) of the interference mitigation game. The learning algorithm is based on the fact that an UAV, suffering from intra-cluster interference, is randomly chosen to update its joint channel-slot selection according to its experienced interference level, slot interval and cluster rewards in each step and stochastically determines the channel selection. The simulations focused on convergence behavior, selection behavior, and performance evaluation, outlining the importance of setting appropriate weights for improved interference management by the log-linear algorithm. In this way, the proposed algorithm converges fast to achieve the optimal network utility and the minimum weighted interference level.

Interference mitigation in networks where both ground nodes and UAVs are connected through a cellular is studied in Reference [44]. The goal of this study is twofold: first, to develop a path-planning scheme for the UAVs taking into consideration the interference that is introduced to the ground nodes and, second, to minimize the latency of the data related to their flight plan. So, an ESN-based DRL framework for online UAV trajectory optimization is presented and a noncooperative game is formulated consisting of the UAVs as the players and having the objective of learning their paths and of transmitting power level and cell association in an autonomous manner. Moreover, to overcome the hurdle of knowing the entire ground topology, the UAVs adopt the RL ESN-based algorithm to predict the ground network dynamics and pto lan their trajectories and resource allocation. Performance evaluation showed that the proposed approach is able to reduce the wireless latency for the UAVs and to increase the rate for the ground UEs at a complexity that is comparable to the conventional shortest path scheme.

Then, the paper in Reference [45] proposed the use of LTE-Unlicensed (LTE-U) for deploying UAV BSs to establish wireless connectivity after a natural disaster. In such settings, coverage is limited due to damages of the ground LTE infrastructure while Wi-Fi connectivity might be possible in some areas. Thus, this study focused on interference management between LTE-U and Wi-Fi transmissions. As a result, deriving the optimal user association strategy between these networks is crucial and this problem was formulated as a game between the Wi-Fi access points (APs) and LTE-U UAV BSs. The UAV BSs aim to maximize their capacity without maximizing the interference of the Wi-Fi network. Thus, the LTE-U transmission period was configured by employing a regret-based learning dynamic duty cycle selection technique, maintaining a balance for the throughput of both networks. The performance evaluation results revealed that, in coexisting LTE-U UAV BSs and Wi-Fi APs topologies, the learning-based duty cycling improved the throughput by 32% when compared to fixed LTE-U duty cycling while an improvement of 10% was achieved over Q-learning-based duty cycling.

### 2.3. Transmission Parameters Configuration

In order to maintain reliable communication and efficient utilization of wireless resources, optimal configuration of the transmission parameters should be performed. The study in Reference [46] focuses on adaptive modulation and coding (AMC), proposing a heterogeneous deep model fusion (HDMF) method. The problems of feature extraction and classification are solved through the adoption of DL while obtaining filters based on learning for reducing computational complexity without compromising classification accuracy. Training data are acquired through a real geographical environment forming the basis for the classification tasks. In greater detail, various single-carrier modulation samples with additive white Gaussian noise (AWGN) and multi-path fading are generated for various signal-to-noise ratio (SNR) values. Then, CNNs and long short-term memory (LSTM) are used for time-domain processing of the modulation signal’s waveform. CNNs and LSTM are combined accordingly in serial and parallel modes tackling the AMC problem, thus resulting in two HDMFs. Both HDMFs are trained in order to learn the transmission features and to make appropriate classifications to determine the employed AMC. The results show that HDMFs exhibit improved performance compared to single CNN or LSTM for SNR ranging between 0–20 dB.

In massive multiple-input multiple-output (MIMO) systems, precoding matrices are essential to improve the performance of the transmission by exploiting the channel state information (CSI). As UAVs can be repositioned in order to achieve LoS conditions with ground users, they are considered as an enabling technology for mmWave communications, and the construction of the precoders can be viewed as a training process. In Reference [47], a hybrid precoding architecture based on a lens array for UAVs was proposed, enabling high efficiency at a low cost. Therefore, a robust and energy-efficient hybrid precoding architecture is developed, using the ML cross-entropy (CE) and the relative error estimation optimization methods. In the initial phase, the hybrid scheme randomly generates a number of predicted analog precoders according to the probability distribution, measuring their achievable sum-rates based on the traditional CE algorithm. Then, the predicted precoder is weighted according to the achievable sum-rates. Simultaneously, the relative error between the updated prediction of the precoder and the near optimal probability distribution is calculated, while the relative error is being updated. In the final phase, an analog precoding matrix close to the optimal probability distribution is calculated. Simulation results outline that, even though the proposed algorithm results in reduced sum-rate compared to full digital precoding, due to imperfect system architecture and system array gain losses, it can provide close to optimal performance with low-cost hybrid precoding. At the same time, the hybrid precoding scheme is a feasible alternative for reduced energy consumption compared to full digital precoding, antenna selection, and conventional CE solutions.

Table 2 includes various communications aspects that have been examined in relevant works and respective ML solutions that were followed to enhance their performance.

## 3. Security and Safety Issues

UAVs are foreseen to play a major role in various domains of 5G and beyond networks, supporting not only improved communication but also safety applications and critical infrastructures. Moreover, the tremendous growth of the UAV market will result in numerous heterogeneous air-to-ground deployments with increased densities of UAV nodes, further necessitating their protection against cyber-attacks across different network layers.

### 3.1. Physical Layer Security (PLS)

PLS has been proposed as an efficient way of safeguarding the operation of wireless networks against a variety of attacks, ranging from jamming to passive and active eavesdropping [48,49]. More specifically, PLS complements encryption solutions through the exploitation of information theoretic methods in order to enhance the secrecy level of wireless transmissions [50]. Focusing on UAV networks, various studies have investigated the application of PLS solutions. In Reference [51], a UAV is employed to perform a smart attack against a source–destination pair. The UAV is capable of overhearing the transmission of the source while generating a spoofing or jamming signal to reduce the quality of the destination’s reception. In order to evaluate the impact of practical assumptions, imperfect channel estimation is considered due to limited pilot signals. In this setting, a secure communication game is formulated and channel estimation errors as well as the UAV smart attacks are jointly tackled through Q-learning. By exploiting the transmission history data, transmit power adaptation is performed to enhance the secrecy of the transmission. In addition, the NE strategy of the noncooperative game under the channel estimation errors was developed, maximizing utility function of the source while mitigating the effect of the UAV smart attacks. Simulation results illustrated that the proposed Q-learning-based strategy enhances the PLS performance, decreasing the attack rate regardless of the channel estimation error. Nonetheless, in order to guarantee the convergence of the learning algorithm, knowledge of the action space of each player is required.

Next, the issue of smart attacks on UAV-enhanced vehicular ad hoc networks (VANETs) has been studied in Reference [52]. There, a UAV acted as a relay for forwarding the messages of an on-board unit (OBU) to a roadside unit (RSU) when the latter experiences severe interference or jamming. The UAV–smart jammer interaction resulted in an anti-jamming UAV relay game, where the UAV determined its relay decision towards a different RSU and, at the same time, the jammer observed this strategy and selected an appropriate jamming power level. The NE of this game was derived showing the dependence of the optimal relay strategy on the transmission cost and the air-to-ground channel model. For the selection of the optimal relay strategy against jamming, a hotbooting policy hill climbing (PHC)-based learning approach was designed without requiring knowledge of the UAV channel model and the jamming strategy. Simulation results revealed that PHC-based relaying can reduce the bit error rate performance of the VANET compared with a scheme based on Q-learning.

Further research on security aspects of coexisting VANETs and UAVs has been conducted in Reference [53]. In greater detail, prospect theory (PT) was used to formulate a subjective smart attack game for the UAV transmission, in which a smart attacker selected its attack type from jamming, spoofing, and eavesdropping without knowing the attack detection accuracy of the UAV system. Simultaneously, the UAV transmit power across different channels was appropriately set to resist these smart attacks. Probability weighting and value functions were applied by PT in order to model the subjective decision-making processes incorporating the fact that people tend to avoid risks due to incurring losses. Then, RL-based UAV power allocation was proposed to safeguard the transmission against smart attacks without being aware of the attack model and the channel model. Performance evaluation indicated that the proposed UAV power allocation method can decrease the impact of smart attacks and can increase the secrecy capacity of the UAV transmission by 16% and the utility by 22%, as compared with the Q-learning based scheme.

Protection against global positioning system (GPS) spoofing attacks on UAV networks was the subject of the paper in Reference [54]. As fake signals can confuse the UAV and the air traffic controllers, mitigating the impact of this attack type is vital for UAV systems. Thus, a supervised ML approach based on ANN for detecting the GPS spoofing signals was developed. In order to classify the GPS signals and to train the ANN, characteristics such as pseudo range, Doppler shift, and SNR were acquired. An important feature of this solution is its applicability in current UAV systems, as it does not require any modification of the GPS equipment. The results illustrated that the ANN-based solution exhibits high detection probability of spoofing signals and reduced false alarm probability, compared to the stochastic gradient descent (SGD), searching for the minimum using conventional Newton–Raphson iterative solving based on a subset of samples and the adaptive moment (Adam) estimation employing dynamic per-parameter learning rate to calculate the minimum of the cost function.

Then, the potential of using unsupervised learning for the detection of active eavesdropping in UAV-aided relay-based networks was investigated in Reference [55], where the uplink was responsible for the authentication procedure. More importantly, one-class SVM and K-means clustering were exploited for the detection of possible attacks during the authentication. To create training data sets for the ML models and to facilitate the training process, the wireless signals and the statistical knowledge of CSI were used. According to the results, the one-class SVM was more stable than k-means. However, k-means is preferable, provided that the eavesdropper’s transmitted power has a sufficiently high value.

### 3.2. Public Safety Applications

Due to the flexible deployment of UAV networks, several public safety applications rely on flying nodes, acting as BSs for establishing communication after a disaster or as monitoring stations for assessing the condition of critical infrastructures and environmental attributes. In Reference [56], a defense system for tackling malicious UAVs was proposed. The defense system employed various UAV detection techniques, such as jamming, GPS spoofing, transmission of hacking signals, and laser shooting. Then, its interaction with malicious UAVs was cast as a dynamic defense game, where the appropriate defense policy was chosen, considering the attack mode of the UAV and the significance of the protected area. Towards this end, the author adopted the Markov decision process (MDP) framework to model the defense policy selection. Also, Q-learning is used to determine the optimal defense policy without requiring any prior knowledge of the current attack mode of the UAV in the dynamic game. The simulations that were conducted indicated that the proposed RL-based malicious UAV interception scheme reduced the risk rate of the protected area and improved the defense system’s utility when compared to a random defense policy.

Then, in disaster mitigation and management scenarios, where rapid changes in the environment usually occur, UAV swarms can be employed for effective and real-time mapping of complex and large-scale zones. In this regard, ML-based modeling and simulation techniques for an UAV swarm system with autonomous capabilities were presented in Reference [35] and the decision-making procedures were distributed among all the swarm members. In particular, an efficient mapping procedure based on genetic algorithms searching on a population of potential solutions was proposed to optimize the information gathering and to minimize the percentage of unmapped sites in the area of interest.

Next, the work in Reference [57] focused on the issue of UAV hijacking and compared various ML approaches to analyze and classify the behavior of different UAV pilots. To generate data, twenty UAV pilots were employed to fly the same UAVs in three specific trajectories. Pilot identification relied on the way that each pilot controlled the UAV and not on their specific actions, i.e., the structure, cadence, and timing of command patterns. By assuming that the pilots had to carry out identical maneuvers and lossless recording of their behavior, fast identification was possible using five ML classifiers, i.e., linear discriminant (LD), quadratic discriminant (QD), SVM, KNN, or RandF. From the comparisons, it was concluded that the RandF classifier provided the best accuracy at around 90%, exploiting simple time-domain features. More importantly, the classification exhibited low complexity, and thus, it was suggested that it can be performed on-board the UAV for achieving real-time interception and prevention of hijacking. However, the accuracy is strongly related with the pilot and the flight trajectory, as well as the pitch, roll, and yaw effects. Moreover, thrust control signals exhibit varying levels of significance for pilot identification.

In addition, the authors of Reference [58] aimed at integrating UAV surveillance capabilities with a cloud-computing environment and an appropriate user interface (UI) for efficient emergency response. More specifically, the authors assumed limited computing and energy capabilities at the UAV and, so, computation offloading of the acquired data was performed at the cloud. Security issues in this scenario are related to the interception by malicious nodes of the UAV data, threatening their privacy, confidentiality, and integrity. In order to overcome such concerns, the end-to-end solution is based on on-site video data collection by the UAV, which employs a keyframe selection algorithm for video compression and latency reduction. Moreover, secure wireless communication is established using a secure component at the UAV, enabling the cryptographic protocols of the compressed video stream. Then, initial real-time evaluation of the collected data is performed at the cloud and CNN-based detection is developed for achieving an improved trade-off amongst detection accuracy and latency. The performance of proposed solutions is evaluated on a hardware platform based on an Infineon Larix drone and a Raspberry Pi connected to a physical secure element (SE) responsible for the cryptographic protocols, a camera, and a graphics processing unit (GPU) server. Performance evaluation showed that secure communication, fast detection, and a user-friendly UI can facilitate the emergency response in civilian applications. Moreover, a trade-off between an accuracy above 65% of the mean average precision and a processing time below 150 ms can be achieved when data processing relies on the Kullback–Leibler (KL) divergence metric instead of root mean square distance (RMSD).

Another critical application was examined in Reference [59], aiming to overcome privacy and public safety concerns arising from the use of UAVs by amateurs over sensitive areas. Towards this end, a distributed system was proposed for identifying and locating dangerous UAVs based on wireless acoustic sensors and SVM-based ML algorithms. Then, the plethora of RF characteristics and telemetry data of amateur UAVs was analyzed using a software-defined radio (SDR) platform adopting a SVM-based ML approach, deriving classifiers to specify if a transmitted packet came from an UAV. After the telemetry protocol was decoded, the system transmitted control commands to the UAVs and, if they insisted on their malicious behavior, aggressive surveillance UAVs were deployed. From the simulations, it was suggested that the proposed ML-enhanced SDR platform enables efficient and proactive protection of the sensitive area against trespassing UAVs. More specifically, the acoustic detection performance of UAVs adopting the SVM-based solution is satisfactory but demands relatively high signal-to-interference ratio (SIR). Also, the acoustic location performance is severely affected by Gaussian noise and erroneous signal latency measurements. Finally, the identification of a telemetry data packet presents significant fluctuations, depending on the bit error rate.

Finally, the study in Reference [60] proposed the deployment of low-cost surveillance UAVs equipped with smartphones for small boat detection, which is of high importance in search and rescue as well as border patrol operations. More specifically, the UAVs transmitted images using the cellular infrastructure to a distant cloud server. In the cloud, a CNN was trained and detected various classes of small boats as well as people in order to trigger security alerts. Moreover, the proposed system included an automatic identification system (AIS) for comparing images of the detected boats and for comparing its GPS position with a marine traffic database, classifying it either as a registered or unregistered boat. For enhanced security of the signal transmission, various encryption mechanisms were integrated in the system, guaranteeing data integrity while jamming attacks were mitigated through Gaussian noise overlapping. More importantly, the proposed light NN offers up to 99% accuracy of object detection, greatly facilitating the tasks of search and rescue as well as border patrol operations.

Table 3 summarizes UAV security issues that were investigated in the literature and the corresponding ML solutions that were adopted to address them.

## 4. Resource Management and Network Planning

In the novel 3-D and dynamic architecture that is introduced in the UAV-cellular networks, resource management, network planning, content-caching, and user association tasks are very demanding, since many contradictory requirements should be considered, such as low latency, increased throughput, low overhead, support of massive number of devices, and dynamic conditions. In that sense, the ML framework has been adopted to facilitate resource management in a quite efficient manner. In Reference [61], the authors aimed to predict the success and failure rates in an UAV network by using ML algorithms based on linear regression (LR) and SVM. Since UAV connectivity is time variant due to their mobility, the success probability of the transmission decreases as the wireless links’ distance increases. In this setting, ML methods can train the UAVs to determine whether connectivity with neighboring nodes can be achieved. Simulations revealed that accurate training by LR and SVM can be attained with SVM offering improved precision and speed. Through timely training, multi-UAV networks can be optimally deployed and accurate clustering can increase the reliability of wireless transmissions.

In Reference [62], ML techniques have been applied to predict cell quality for UAVs that are connected to a cellular network. More specifically, a new conditional random field position is proposed for predicting the best serving cell for one node at a given location. The main idea is to exploit the spatial correlation that naturally exists in aerial channels nearby positions. For evaluating the performance of the proposed approach, real 3GPP LTE simulation parameters have been assumed for the cellular model. The numerical results have shown that the proposed scheme offers high accuracy and improved performance as compared to other heuristics methods.

Then, in Reference [63], UAVs and device-to-device (D2D) communications are jointly employed to recover the connectivity in a disaster area. To this aim, two UAVs have been used for serving as aerial BSs. The goal is to select the users to be served by the two UAVs (or via D2D connections) that maximize the weighted sum-rate. This user association problem can be seen as a clustering one, and thus, a k-means based algorithm has been proposed in order to solve this type of unsupervised learning problem. In this context, two algorithms are given, i.e., a learning-based clustering algorithm and a relaxed optimization algorithm. The numerical results have shown that the learning-based algorithm achieves a relatively high performance close to the benchmark relaxed optimization algorithm at a much lower computational complexity.

In Reference [64], an RL algorithm is proposed for reducing unnecessary handoffs in UAV-based networks. In addition, a k-means method is also adopted as a mobility control approach. The simulation results have proved that the coexistence of the RL approach with the k-means algorithm can effectively reduce the handoffs by 75% and can simultaneously increase the system throughput compared to UAV mobility control based on SNR estimation. Furthermore, in Reference [65], a cellular network is considered that consists of a set of UAVs and a set of ground BSs, which both may serve the users. The main objective is to predict traffic congestion in order to deploy temporary UAV BSs with minimum requirements regarding the communication and mobility powers. This offloading strategy is achieved by introducing a Gaussian mixture model that is based on a weighted expectation maximization algorithm. Performance evaluation has shown that the proposed ML framework can reduce the requirements for the downlink communication power by 20% and for the mobility power by 80% as compared to UAV deployments without the benefits of ML.

In Reference [66], an UAV-enabled LTE-U network is assumed in which the UAVs are able to access both the licensed and unlicensed bands. The goal of this work is to develop an efficient resource allocation scheme that will maximize the QoS and simultaneously satisfy the delay requirements. To this aim, an ESN algorithm has been proposed with leaky integrator neurons, which enables the UAVs to adopt the environmental dynamics. The simulation results showed that the proposed approach achieves up to 14% and 27.1% QoS gains for the UAV associated users, compared to Q-learning using LTE-U and Q-learning using LTE. Such gains are attributed to the dynamic adjustment of the ESN’s state update speed, thus enabling the UAVs to learn the dynamics of their associated users.

In Reference [67], in order to enable efficient multi-modal, multi-task offloading, a new architecture is proposed for UAV clustering. For facilitating UAV cooperation and joint optimization of the computation, caching, and communication resources, an AI-based decision making framework has been proposed in terms of the RNN. To this aim, UAV cluster deployments have been employed, which are based on both historical data mining and real time perception. The numerical results presented have shown that the proposed strategy can effectively improve the collaboration between the UAC clusters.

Moreover, in Reference [68], a cluster-based network has been assumed, where multiple UAVs perform various real-time time tasks, e.g., sensing and transmitting. It is noted that the communication part is based on orthogonal frequency division multiplexing (OFDM). In this framework, RL has been used to solve the allocation problem of wireless channels in dynamic conditions, targeting the optimization of the time delay of UAV data transmissions. The numerical results presented showed the applicability of the proposed framework for real-time UAV scheduling. In addition, RL does not rely on accurate models, thus making it a well-suited solution for UAVs with limited processing capabilities while relaxing the necessity for large offline data sets, as it is the case in supervised learning approaches.

In Reference [69], a scenario where a licensed primary user (PU) shares its spectrum with a network of UAVs is considered. In this scenario, the UAVs can be used as relays for the primary network or can perform data transmission to the fusion center. The objective is to maximize the total utility of the PU and UAV network. To satisfy this objective, a deep distributed reinforcement learning (DDRL) algorithm is employed by which the UAVs chose their role without requiring exchange of information or the knowledge of other’s UAVs decisions. The presented numerical results verify the convergence of the proposed algorithm as well as the maximization of the network resources.

Various works leveraged swarm-based optimization tools in UAV networks. In Reference [70], a network with multiple high amplitude platform stations (HAPS) is considered for communication coverage purposes. The coordination of this network has been performed with the aid of RL and swarm intelligence. It was shown that the RL approach achieves higher overall peak user coverage rates with the cost of slower convergence and the risk of occasional dips while the swarm intelligence-based solution resulted in lower coverage peaks and improved coverage stability and convergence. Next, the authors in Reference [71] proposed and applied network-based heterogeneous particle swarm optimization (NHPSO) in an air-to-ground downlink communication scenario, where multiple UAVs were deployed to provide wireless connectivity to a set of quasi-stationary heterogeneous users uniformly distributed in a geographical area with different required data transfer rates. The goal of NHPSO learning algorithm was to find the global optimum of a complex radio coverage problem while maximizing users’ total required data rates. This was obtained by simulating the swarm in real-world complex systems following a heterogeneous learning strategy of particles along with the heterogeneous network topology structure.

A typical manned and unmanned (MUM) airborne network consisting of both manned aircrafts (in higher layer) and UAVs (in lower layer) forming dynamic swarm network topologies was considered in Reference [72]. To find out the optimal link between two UAV relay nodes that maximizes the throughput performance, a DQN buffer-based model having the ability to store the network topology changes was proposed and the link selection was realized in the aircrafts (control nodes), which could accomplish heavy computational tasks in real-time. Also, an optimization algorithm was designed and implemented at the UAVs to handle the optimal placement of one or more UAVs as relays, under traffic QoS constraints and time-varying link conditions. The results pointed out that the DQN-based optimization algorithm surpassed the Q-learning algorithm.

The uplink wireless channel of an UAV-aided NOMA, energy-efficient, and self-adaptive public safety network (PSN) with clusters of UEs was considered in Reference [73]. The role of UEs, i.e., either clusterheads having a critical role within the PSN or cluster members, was autonomously determined in a distributed manner based on the minority games (MG) theory [74] and Q-learning. To form the clusters, the cluster members acted as stochastic learning automata choosing their clusterhead based on RL and considering their physical proximity and the energy availability. After the cluster formation, the 3-D placement of the UAV was optimized in order to maximize the clusterhead’s energy availability. The optimal transmission power (unique Nash equilibrium) of each UE is determined using a noncooperative game-theoretic method. In the extensive numerical results, the energy efficiency and the effectiveness of the proposed distributed decision-making method were validated, outlining its importance for the efficient operation of PSNs, where often centralized coordination might be unavailable.

Recently, a novel paradigm has been proposed in wireless networks, i.e., content caching at network nodes towards minimizing the backhaul/fronthaul load and reducing the transmission latency. Due to their flexibility, UAVs have been considered to exhibit a pivotal role towards improving the performance of cache-enabled networks. The authors of Reference [75] exploited user-centric information related to content request distribution and mobility patterns for deploying UAVs and for determining content caching on their buffers. The optimization targets were to maximize the QoE and to minimize the total transmit power used by the UAVs. Assuming that cloud computing provides the necessary resources for the accurate prediction of content and user parameters, the optimal caching strategies are provided to the UAVs. The individual user behavior is classified by the cloud into distinct patterns through a conceptor-based ESN method, enabling improved accuracy through ML-based prediction. As a result, the ESN-based method facilitates the extraction of the user-UAV association, UAV placement, and content caching strategy. The simulations reveal that the UAV transmit power can be adequately reduced compared to an algorithm without caching as well as conventional ESN approaches, while QoE is significantly increased compared to a topology without UAVs.

Then, focusing on a LTE cloud network operating in licensed and unlicensed bands, resource allocation for cache-enabled UAVs supporting ground users was studied in Reference [76]. Constrained by the limited capacity of the UAV-cloud links, LSM is employed to help the UAVs perform content caching and resource management. LSM enables the cloud to efficiently learn user-centric information regarding content request distribution and to facilitate spectrum allocation by the UAVs. This method is extended in Reference [77], where an optimization problem is formulated to maximize the number of users with stable queues. As a solution to this problem, a self-organizing, decentralized algorithm is developed and LSM is employed for joint caching and resource allocation over both licensed and unlicensed bands. Then, performance evaluation is conducted illustrating the increase in the number of users with stable queues in comparison to Q-learning with and without content caching. In a similar setting, with cache-enabled UAVs, the paper in Reference [78] introduced proactive caching for latency and backhaul load reduction. Thus, a multi-objective optimization problem is formulated targeting parameters, such as minimum number of deployed UAVs, transmit power, UAV-user association, and cache location. To solve the optimization problem, RL is adopted for user grouping, performing local search according to the optimal UAV deployment over each group. From the results, the efficiency of the RL method is shown, effectively minimizing the number of required UAVs and their 3-D placement in the network.

Table 4 summarizes all the resource allocation and network planning techniques which employ ML algorithms for UAV-enabled communication networks.

### 4.1. Energy Efficiency and Power Control

UAVs have limited capabilities in terms of energy resources, which makes the energy and power consumption optimization a very important factor of the overall network’s performance [79]. In that sense, various ML solutions have been proposed that exploit in a near optimal manner the network’s resources towards minimizing energy consumption. In Reference [80], a group of UAVs is considered to fly at a certain altitude that allows them to provide communication coverage for ground users in a specific region. The main objective of this paper is to achieve a certain communication coverage, while preserving the UAVs’ connectivity and simultaneously minimizing their energy consumption. More specifically, a DRL method is proposed for controlling the UAVs in order to maximize the energy efficiency, while ensuring effective and fair communication coverage and network connectivity, learning and adapting to the wireless environment by relying on two powerful deep NNs. In the simulations, the proposed approach was compared to two conventional methods without ML capabilities. The first one, titled Random, randomly selected a moving direction and a flying distance for each UAV, while the second one, titled Greedy, selected the moving direction and flying distance in a sequential manner, maximizing the instantaneous reward for every UAV abiding to the region boundary and connectivity constraints. It was shown that the ML-based solution provided improved performance in terms of average coverage score, fairness index, average energy consumption, and energy efficiency.

The study in Reference [81] presents a holistic framework for PSNs based on UAV deployment that communicates through NOMA and recharges the batteries of IoT ground nodes under the wireless powered communication (WPC) paradigm. The main motivation behind this work is to adopt ML in order to address the network lifetime maximization of PSNs and their lack pf centralized cellular connectivity, thus providing a distributed, autonomous, and resilient communication deployment. More specifically, each IoT node selects a specific role, either acting as a coalition head or coalition member in a distributed manner by adopting the MG theory. Moreover, communication can take place either between IoT nodes or with an UAV/evolved NodeB (eNB), while RL is employed to form coalitions of IoT nodes, considering energy consumption, in an autonomous fashion. Then, outlining the importance of the coalition heads operating as gateways for the rest of the IoT nodes, resource management optimization is performed targeting efficient RF energy harvesting, determining the UAV trajectory and uplink transmit power control towards energy-efficient transmissions. The performance of the proposed framework is evaluated via simulations, and it was observed that gains can be achieved in terms of energy efficiency, robustness, and scalability. In Reference [82], a similar system model to Reference [81] was assumed, in which UAVs are used to wirelessly charge a large number of energy-constrained IoT devices; ML can enhance the performance of energy transfer and sensor network communication. These devices are assumed to be randomly distributed in a two-dimensional area, and an UAV is responsible for transmiting energy through RF signals. Thus, the performance of the wireless power transfer and peer-to-peer sensor communication towards a fusion center was jointly considered. Aiming to enhance the performance of both aspects, an ESN-based scheme for sensor energy consumption prediction was adopted. Exploiting these predictions, the distributed sensor nodes were clustered using an improved k-means clustering algorithm under a specific rule to minimize packet losses. Furthermore, the distributed power control problem towards sensor interference mitigation was modeled as a mean field game (MFG), considering both the sensors’ energy and their mutual interference. MFG models the interactions among the behavior of an individual player and the aggregate behavior of the collective players. The numerical results indicated that the proposed schemes can improve the efficiency of wireless charging, being practically applicable due to their offline operation, solving the MFG and thus promoting their practical implementation without demanding significant processing power from the UAVs. Moreover, when compared to a uniform power control policy without ML, the signal-to-interference plus noise ratio (SINR) threshold imposed by the network is not violated, thus providing resiliency against outages.

### 4.2. Multiple Access and Routing Protocols

The UAV-enabled networks are a new communication paradigm that is characterized by high-speed mobility and frequent topology modification. In that sense, the traditional multiple access and routing protocols are not able to fully support the highly dynamic nature of the flying ad hoc UAV communication networks. A promising approach that could improve this aspect is to adopt ML in multiple-access and routing protocols, leading to autonomous network coordination and distributed user scheduling and data routing through dynamic path formation. Towards this direction, in Reference [83], a demodulation-free medium access control (MAC) identification scheme has been proposed that includes an ML classifier. In particular, SVM has been utilized to identify MAC protocols without demodulation. The proposed approach has been implemented with universal software radio peripherals (USRPs), while its performance has been evaluated using two typical MAC protocols, i.e., time division multiple access (TDMA) and carrier sensing multiple access with collision avoidance (CSMA/CA). In the presented experimental results, an access accuracy rate above 95% was observed while demodulation-free algorithm without ML provided an accuracy rate below 60% in a scenario with dynamic variation of the SNR. In Reference [84], the system model consists of several flying UAVs, which are equipped with a GPS and an identical switched beam antenna array. For improving the capacity of the network, an RL-based routing protocol is proposed, in which all UAVs are able to exchange status information in order to discover the routing path that the shortest delivery delay. The proposed solution enables self-optimization and autonomous operation, establishing connectivity through the optimal routing path. In this way, the average network delay can be reduced, as routing path formation is independent of the often unpractical a priori knowledge requirements, thus offering self-learning through RL. The simulation results showed that the proposed protocol provides improved performance, as compared to the existing ones and can guarantee successful data delivery with low latency.

In Reference [85], an adaptive MAC protocol has been proposed in a scenario with multiple UAVs, where in each slot, the UAVs determine which MAC protocol, between TDMA and CSMA/CA, are more appropriate. A distributed Q-learning-based MAC scheme is utilized in order to preselect the MAC protocol according to the UAV’s current state. The MAC switching decision is based on a practical byzantine fault tolerance procedure. Using this approach, each UAV is able to evaluate its performance and to determine the more appropriate MAC protocol that should be employed. In the performance comparisons, the proposed solution is evaluated against three alternative schemes. The first scheme was a distributed adaptive MAC protocol, allowing the UAVs to select the MAC protocol based on a specific performance metric. The second scheme was a MAC protocol relying on performance thresholds, and if they are violated, switching to another MAC protocol occurs. The third scheme consisted of a MAC protocol without consensus where transmitting UAVs adopts a different MAC protocol until their packet is successfully received by the receiving UAVs that might rely on another MAC protocol. Extensive simulation results have proved that the performance of the proposed scheme with respect to the average throughput, delay, and packet retransmission ratio is improved as compared to other conventional MAC protocols. In Reference [86], assuming a decentralized edge UAV swarm, a multi-armed bandit (MAB)-based approach has been proposed for optimally selecting the energy-efficient and processing data offload paths between a source and a target UAV. A large-scale UAV swarm emulator was implemented in Python that was based on real-life hardware metrics collected by UAVs in flight. The performance results showed that the proposed MAB approach provides more energy-efficient and processor-friendly path solutions as compared to other solutions.

In Reference [33], a hierarchical architecture has been considered, in which UAVs coexist with low earth orbit (LEO), medium earth orbit (MEO), and geostationary earth orbit (GEO) satellites. In the scenario under investigation, shortest path routing will result in higher priority to MEO satellite routing, which, however, does not guarantee improved performance due to the periodically burst traffic. In this context, it was proved that a traffic balance is achieved, by adopting a DL approach for transferring some traffic to GEO satellites, with a reduced computational cost if distributed periodical training is used. The numerical results presented have showed that the DL approach increases network throughput as well as reduces packet loss rat, as compared to other routing schemes, especially in scenarios with large number of source nodes. This performance gain derives from the fact that the proposed DL architecture learns from traffic patterns and transmits some of the packets through GEO satellites when a number of MEO satellites experiences congestion. At the same time, the conventional strategy firstly selects the MEO satellites for packet transmissions, often resulting in congestion in the MEO satellites.

## 5. Position Related Aspects

One of the major challenges of designing UAV-based communication systems is to determine the proper horizontal and/or vertical placement of the UAVs as well as the trajectory of the UAVs with regard to other ground or flying objects in order to achieve optimal or near-optimal performance. Moreover, in critical and forensics applications, where public safety and security are of high importance, the rapid and efficient physical detection, localization, and identification of the type of noncompliant UAVs is indispensable [87]. Next, we survey works related to detection/localization as well as placement and trajectory design.

### 5.1. Detection and Localization

The detection of UAVs can be achieved either by direct self-reporting or indirect discovery performed by ground BSs. Conventional detection techniques, including radar, LiDaR sensors, acoustic recognition, electro-optical sensors, and computer vision seem inappropriate in challenging overcrowded dense-urban environments with numerous physical obstacles, ambient noise and light variation, and NLoS propagation conditions. To overcome these issues, intelligent detection and identification techniques have emerged based on data driven algorithms. Among them, ML classifier models have been previously proposed as an accurate classification method. Moreover, DL methods have also been used for the UAV detection problem.

In Reference [88], a complementary ML-based identification method was applied using large training data sets consisting of UAV and non-UAV data flows from real-world encrypted Wi-Fi traffic. In general, exploiting Wi-Fi traffic is not straightforward due to possible data encryption whereas applying ML methods for real-time UAV identification may not always be beneficial due to time constraints and possible processing delays. Thus, a delay-aware ML-based UAV identification framework was proposed that achieves permissible accuracy while minimizes the delay. This framework handles the encrypted data flow as a time series, exploiting information of packet size and inter-arrival time for statistical feature extraction and estimating the packet inter-arrival time using maximum likelihood estimation (MLE) method. The prediction time is significantly reduced due to l1-norm regularization and the integration of the feature selection and accuracy optimization in one objective function. Based on the results, the delay-aware ML solution achieves an accuracy of about 85–95% being capable of identifying UAVs within 0.15 to 0.35 s in four scenarios where, in each one, a data set from a different traffic class was used.

In Reference [89], an air-to-ground LTE cellular network was considered, under strong LoS propagation conditions and harsh interference. The existence of the aerial users (AUs) was accurately and timely detected using ML and measured RF data from UEs as input in a rural area. More specifically, for the AUs detection, a relatively small number of training samples and a weighted distribution for the training set were used along with three ML techniques: (i) the rather simple and insensitive to the distribution or size of the training set Bayesian estimator [90]; (ii) the SVM with high computational complexity and high sensibility but adequate performance [90]; and (iii) the MLP [91], which supports real-time on-the-fly training and avoids storage costs. It is noted that the decision time and the performance of the classification algorithms are a function of the number of features. Using the proposed ML classification methods and an significant number of features, the reliability was approximately 99% whereas the accuracy approached 100%. Moreover, it was observed that, depending on the available data set, each ML solution can lead to different results, as it is the case with unevenly balanced data among aerial and ground users. More specifically, Bayesian and SVM estimators exhibit better generalization capabilities for different performance metrics, while MLP can offer improved performance if a specific metric is targeted.

An ANN-based detection algorithm was proposed in Reference [92] using three features: the slope, the kurtosis, and the skewness of the signal received from an UAV. Both outdoor and indoor experimental measured data from RF signals were used to train the algorithm and to obtain feature extraction and classification of UAV or non-UAV signals. The results in terms of the error rate revealed that the recognition rate can exceed 82% within a distance of 3 km, a score that is better than other scores provided by conventional non-ML detection methods. The detection and classification of UAVs using Markov-based naive Bayes ML techniques was also realized in Reference [93] for multiple RF raw signal fingerprints from several UAV controllers and different SNR levels. In particular, the classification was based on the energy transient signal and statistical processing in order to avoid noise sensitivity and to adapt to modulation techniques. This method is characterized by low computational complexity, does not exploit the time-domain, and hence averts possible delays for the detection of the transient of the signal, especially in low SNR scenarios. The feature sets were used to train different ML algorithms, including the KNN classification [90], the discriminant analysis (DA), the SVM, and the NN. The results showed that the KNN classifier has the best performance, obtaining an accuracy of above 80% for 15 dB SNR and 14 controllers, whereas the NN has the the worst performance when SNR is low. Further, increasing the number of controllers leads to an average accuracy of 96.3%. Overall, RF fingerprinting is a promising technique, but it requires specific equipment, e.g., expensive SDRs at high frequencies.

In Reference [94], an acoustic ML-based UAV detection approach with multiple listening nodes, i.e., computing units capable of detecting the UAV sounds, and a control center was presented, relying on features such as Mel-frequency cepstral coefficients (MFCC) [95] and short-time Fourier transform (STFT) for training. STFT provides more signal information but contains more noise compared to MFCC, and recently, it has been considered promising for training using acoustic signals, as DNNs and DL are capable of processing more complicated data. SVM and CNNs were considered in order to estimate in real-time the flying path of an UAV in noisy environments. Overall, the STFT-SVM model succeeded in achieving the best performance, as SVM shows improved classification performance in binary tasks with reduced training resources compared to CNN. The current flying status (i.e., whether it is flying or it is static on the ground) of a powered-on UAV that is remotely controlled by a malicious operator was detected in real-time in Reference [96] by eavesdropping the communication traffic exchanged between the UAV and its controller and by applying classification algorithms. In particular, the Weka platform [97] was adopted that contains various standard ML algorithms for data mining tasks. Among them, the Trees-J48 (J48) [98], RandF [99], and NN were used. This cost-effective passive method does not require special equipment or signal transmission and intends to statistically analyze features, such as packet size and inter-arrival time analysis. The results showed that the delay is less than 4 s with 93% detection accuracy.

In Reference [100], a system that autonomously, instantaneously, and accurately locates the controllers of UAVs from the transmitted RF control signals was described. This system uses an RF sensor array to monitor the signal spectrum, whereas a CNN is trained in order to predict the bearing of the drone controller relative to the sensor and, then, estimates the position of the controllers. Using the proposed configuration, an operator can be apprehended at distances of up to 500 m with a mean absolute error of 3.67${}^{\circ }$ in bearing calculation, which in turns corresponds to a fair positional error of 40 m. Then, in Reference [101], ML tools have been used for estimating the locations of drone-UEs based on sparse information. In particular, employing the kernel density estimation, the UAV spatial distribution has been estimated and then used for obtaining the optimal cell association. Moreover, tools from the optimal transport theory have been also adopted. The presented results confirm that the proposed approach significantly reduces the UAVs-UEs latency as compared to conventional UE-cell association based on SINR while also improves the spectral efficiency. Finally, three deep neural networks (DNN) are trained and tested in Reference [102] using an open source RF database in order to detect the presence of UAVs, to identify their type, and to determine their flight mode. This database includes raw RF signals, which were collected for a variety of flight modes. The performance of these DNNs was verified via a tenfold cross-validation process. According to the classification results, the number of classes dramatically affects the accuracy, since some UAVs were manufactured by the same company. In particular, the mean accuracy decreased from 99.7% for the first DNN (2-classes) to 84.5% for the second DNN (4-classes) and to 46.8% for the third DNN (10-classes).

In UAV swarm mission-critical scenarios, each UAV should detect and track the other swarm members in order to acquire information regarding the relative distance and bearing. In this direction, a DL- and visual-based detection/tracking algorithmic framework based on the You Only Look Once (YOLO) object detection system [103] was proposed in Reference [104]. In this framework, the tracker, i.e., UAVs equipped with a low-cost and lightweight visual camera, aims at reliably predicting the location of target UAVs on the image plane of their camera. To experimentally evaluate the performance of this approach and to collect multiple data sets of images to train the learning algorithms, a flight test campaign comprising different UAVs and cameras with varying resolution was accomplished. The results underlined the accuracy of the proposed method, which obtained 90% of correct detection instances, in a timely manner. These results also demonstrated that this method offers robustness against challenging conditions, such as sun illumination, as well as background and target-range variability.

Table 5 summarizes papers tackling detection and localization issues through ML-based techniques in UAV networks.

### 5.2. Placement and Trajectory Design

This challenge is also directly related with the nontechnical but equally important legal and regulatory issues of constructing an international legislation framework for UAV operation [105]. These regulations do not permit UAVs to fly over all areas and introduce altitude limitations as well. Note that there exist some common rules across different countries: (i) the UAVs should fly only in visual line-of-sight (VLoS), usually limited to 500 m from the pilot; (ii) the UAVs should fly only under 120 m relative to the ground. For other operations, such as flying over people, cities, beyond VLoS (BVLoS), etc., special permissions can be granted depending on the country, while testing of new systems and applications must be usually done in segregated airspace areas. Moreover, flying over people not involved in the operation is not allowed in most countries or it is allowed only under a specific authorization of the aviation authorities. Several studies on the optimized placement and trajectory of UAVs as BSs or relays have been previously conducted (e.g., References [106,107]). These studies have focused on providing efficient connectivity to a group of distributed ground users.

The joint optimization of trajectory and power control in multiple UAVs scenarios was obtained in Reference [108] aiming at maximizing the users’ throughput and satisfying the users’ rate requirement. The optimization problem relied on ML techniques and included three steps in order to determine the trajectory design of UAVs acting as agents: (i) a multi-agent Q-learning-based placement algorithm was proposed for estimating the 3-D optimal placement of the UAVs with respect of the initial positioning of the ground users, and (ii) the mobility and the upcoming positioning of the ground users were predicted using an ESN-based prediction algorithm [109] and a real-world geographical data set. The latter one was collected from an online social network, i.e., Twitter, as input and included GPS coordinates of anonymous users and recorded time stamps. The third step of the optimization problem included a multi-agent Q-learning-based algorithm that was used to determine the optimal position and to transmit power of the UAVs in each time slot according to the users’ mobility. The results underlined that the proposed algorithm can converge to an optimal state while comparisons with two benchmark schemes, i.e., the historical average (HA) model and the LSTM depicted the superiority of ESN in terms of MSE over both HA and LSTM models at a lower complexity. These results also illustrated that the proposed approach can increase the throughput by approximately 17%, while accuracy improves, as the size of the reservoir increases.

The authors of Reference [110] follow a Gaussian Process (GP) approach to derive the air-to-ground communication channel, providing an additional link to a group of ground nodes, thus improving their communication quality. For this purpose, GP is employed for predicting the communication channel strength at random UAV positions in an urban environment. Then, the channel model can be used to perform optimal UAV trajectory planning, either with offline pre-scanning based on GP, followed by a nonlinear model predictive control (NMPC) planner or NMPC planner with online GP. More specifically, in the offline method, the UAV performs data measurements from ground nodes via a prespecified scanning pattern flight. After, these data are fed to the GP to build the channel strength between the different UAV positions and ground nodes. On the contrary, in the online method, the UAV trajectory is designed according to the current knowledge of the air-to-ground channel and through periodic measurements by the UAV incomplete map using GP. It was shown that the offline creation of the communication channel strength map, with GP prior to the start of the mission, outperforms the online creation of the map during the mission without scanning.

In Reference [111], the trajectory of an UAV BS employed to provide communication services to multiple users was optimized targeting sum-rate improvement using Q-learning. To model the air-to-ground channel between the UAV BS and the ground users, the log-distance path loss model was used. The simulation scenario included an UAV BS flying at a constant altitude and acting as an autonomous agent, two static ground users, and a cuboid obstacle. To obtain Q-function approximators, a standard table-based approach and an NN were utilized. Based on the simulation results, the UAV BS was capable of learning the network topology without preexisting knowledge of the environment. Also, the UAV BS could autonomously obtain its landing position in a timely manner. These results also underlined that NN is more efficient and scalable and requires significantly less training data than table-based Q-learning.

In topologies where mobile ground nodes require UAV relaying, the paper in Reference [112] proposed to combine ML-based measurement with a probabilistic LAP channel model to facilitate UAV trajectory planning. More specifically, the authors assumed four distinct types of urban environments with unknown building position and shape and a wireless channel including the effects of path-loss, multi-path fading, and shadowing with empirically-known distributions. Also, the ground nodes’ current positions are available to the UAVs but their mobility patterns are not known. Thus, in order to select the appropriate probabilistic LAP model, UAVs collect a pair of signal strength and elevation angle measurements among the UAV and ground nodes. This data allows a neural network (NN) predictor to determine the current urban environment type. The NN’s output can be used for UAV trajectory design using a a Cross Entropy Optimiser (CEO) to generate a set of possible trajectories. Then, a convergence criterion is imposed and, when it is fulfilled, the optimal trajectory is fed to the UAVs. By periodically performing this process, channel prediction for trajectory design in scenarios with mobile ground nodes can be efficiently achieved. Therefore, the NN-based trajectory planning exhibits promising performance in settings where little information on the mission area is available while considering the mobility of the network nodes.

The non-convex nondeterministic polynomial-time hardness (NP-hard) problem of jointly handling in real-time the 3-D deployment and the dynamic movement of multiple UAVs was studied in Reference [113], where the goal was to maximize the sum mean opinion score (MOS) of ground mobile users while attaining an adequate QoE. A three-step solution was proposed, which comprised (i) the use of a genetic algorithm based k-means algorithm [114] to determine the initial cell partition of the users; (ii) the development of a Q-learning based deployment algorithm, which considers each UAV as an agent, trying offline to self-train and find its optimal 3-D placement assuming static ground users (at the first time slot); and (iii) the development of a Q-learning based movement algorithm for scenarios where the users are moving. The results demonstrated that these algorithms rapidly converge to a desired solution after a significantly small number of iterations. Furthermore, Q-learning based-deployment offers improved performance and reduced complexity when compared to k-means and Iterative-GAKmean alternatives.

In Reference [115], an unsupervised online self-tuning learning algorithm for joint mobility prediction and object profiling of the individual UAVs was proposed. Apart from predicting the flying objects’ future locations without requiring prior knowledge of the mobility profiles or trajectories of the UAVs, the proposed method also enables the classification of the UAVs into particular groups based on their motion properties, e.g., rotatory and fixed-wing UAVs, via an hierarchical generative model. From the results, a success rate of 90% in profiling mobile objects was yielded for a reasonable noise level and a relative small training data set (over time) compared to conventional data-driven methods. Overall, this method can be practically applied to FANETs with dynamic network topologies and autonomous UAVs and to predict future topologies.

A UAV-based IoT data harvesting scenario in an urban area was also presented in Reference [116], where a resource-constrained aerial base station was employed to serve multiple static ground nodes, e.g., IoT sensors. Contrary to most works in the field, the propagation parameters are considered to be unknown. Based on this uplink scenario, a joint flight trajectory and node scheduling design problem was formulated to minimize the estimation error of the channel model parameters and to maximize the data traffic between the UAV and each node. The trajectory learning phase included the collection of measured data from the ground users that resulted in adequate knowledge of the propagation parameters from the UAV side. Then, an iterative path planning algorithm was proposed along with dynamic programming [117] techniques and the exploitation of a 3-D city map compression method in order to efficiently handle the aforementioned non-convex problem. The results demonstrated the benefits of the proposed learning method, whereas it was proved that the algorithm can converge to at least a locally optimal solution. Moreover, even though the ML-based solution leverages the rich map data, a map compression method is employed, making the trajectory design problem less complex compared to standard optimization tools.

Then, in Reference [118], a UAV BS flying at an altitude well above the building height was adopted for the provision of video streaming services to several UEs clustered in a circular area. In an effort to optimize the flight planning and, thus, to enhance the QoE of UEs with regard to the video segment delay during streaming applications, a Q-learning approach was formulated. This approach was denoted as Q-SQUARE and modeled the UAV BS’s path as a sequence of states, which were related to the UAV BS’s position, the elapsed flight time, and the residual energy. Also, Q-SQUARE considered multiple recharging stations, where the UAV BSs were recharged after a flight period. The numerical results demonstrated that Q-SQUARE can substantially improve the system performance in terms of the QoE by optimizing the flight path of multiple UAV BSs serving the area of interest.

The joint optimization of the placement and power allocation of multiple UAVs with respect to the ground users in an unknown region was also accomplished in Reference [119]. Since initially, the UAVs have imperfect CSI; the optimization problem was formulated by exploiting game theory. Then, a robust and distributed learning algorithm was proposed to guide multiple UAV flight plans in order to maximize the sum-rate with fairness for all ground users under specific flight region and power constraints. This algorithm converged to a stable state of maximizing the aforementioned optimization objective, resulting in a guide that facilitated the flight path of the UAVs. A flight path planning model for a dynamic and auto-configurable FANET was presented in Reference [120]. This model took advantage of a metaheuristic optimization-based approach that intends to optimize in real time the position of a flying relay device, i.e., an UAV, in order to obtain the best throughput while having knowledge of the position of the other UAVs. Thus, an ANN was trained using mobility data of UAVs including the position and traffic information that was supplied through the Network Simulator 2 (NS-2). According to the results, the average throughput of the optimized FANET was significantly higher, i.e., 135%, in comparison with the non-optimized FANET in an area of 200 × 200 m.

In Reference [121], a Q-learning positioning approach was proposed in an effort to find the best 3-D placement of drones in multiple drone small cells considering propagation areas, where the conventional terrestrial communication infrastructure is not operational or accessible due to a large-scale natural disaster. In these temporary intelligent small cells, the drones have limited resources and the ground users could have distinct requirements in terms of the data throughput and the mobility characteristics. The goal of this approach was to facilitate the construction of an efficient emergency communication network by maximizing the total network radio coverage while attaining robustness against dynamic network conditions, mobility issues, and interference. The simulation results included performance comparison between the proposed Q-learning method and random fixed positioning and circular positioning strategies and depicted that the former outperforms the others, in terms of coverage, QoS, and backhaul throughput.

The performance of a downlink air-to-ground communication system was optimized in Reference [122], where an aerial platform acted as a BS and the ground users were not static. In particular, a low-complexity Q-learning algorithm was used to find, in relatively short processing time compared to heuristic algorithms, the 3-D optimal position of the aerial BS in such a dynamic environment, where the network topology continuously changes, satisfying the QoS requirements. Thus, the benefits of RL-based positioning is evident, as the need for reinitialization in the heuristic-based approaches is avoided and gradual changes due to UEs mobility are efficiently monitored, leading to positioning with lower complexity. In Reference [101], a 3-D cellular network was also proposed that utilizes drones as BSs and drones as UEs. The former use ML tools to determine the spatial probability distribution of the latter for a certain time period, considering the mobility properties and aiming at minimizing the latency. In particular, a kernel density estimation method was developed, for which the training is attained using the sparse (owing to the excessive overhead costs) available prior information of the location of drone-UEs. Additionally, the problem of the proper placement of an aerial platform as a BS was investigated in Reference [123], taking into account the users’ requirements and specific scenarios. Specifically, a DQN algorithm was proposed that maximizes the spectral efficiency by exploiting a large-scale pre-learning experience of different user layouts. The DQN combines the DL CNN and reinforcement learning Q-learning advances, but it has the advantage of being more time efficient. The superior performance of this algorithm was demonstrated in the simulation results, where the spectral efficiency of the system achieved 91.3% maximum spectral efficiency, with lower complexity than conventional genetic algorithms, such as hill climbing and simulated annealing algorithms.

In Reference [124], the self-optimization of multiple UAVs’ trajectory in real-time sensing applications was tackled using a Q-learning learning method in a decentralized manner. A single-cell UAV orthogonal frequency division multiple access (OFDMA) network was considered, where the UAVs transmit the sensory data to a terrestrial BS over orthogonal subchannels to avoid mutual interference and the location of the terrestrial base station and the UAV was specified by 3-D cartesian coordinates. It was also considered that the UAVs perform the sensing tasks in a synchronized iterative manner and send the measured data to the base station, whereas the sensing quality of the UAVs was evaluated using the probabilistic sensing model [125]. In addition, a sense-and-send protocol was proposed that facilitates the coordination of the UAVs handling different tasks, and then, the probability for successful valid data transmission using nested Markov chains was investigated. A frame-level simulation of this protocol was built in MATLAB^®^, and the results underlined the rapid convergence of the proposed algorithm compared to traditional single- and multi-agent Q-leaning algorithms.

A distributed DRL algorithm for the navigation of a group of UAVs acting as BSs flying in a target region was developed in Reference [126]. The optimization problem was formulated as a partially observable MDP (POMDP), where the UAVs were capable of only observing the areas in their vicinity. Since each UAV has connectivity constraints and limited battery lifetime, the optimization problem aimed at improving the temporal average radio coverage and geographical fairness while minimizing the total energy consumption of the UAVs. In this algorithm, an agent, i.e., the UAV, and the environment interact at each of a sequence of discrete timeslots. The training of the algorithm was obtained using the observations of each UAV regarding the propagation environment. According to the simulation results, the proposed optimization method outperfoms the state-of-the-art DRL-EC3 approach based on deep deterministic policy gradient (DDPG) [127] in terms of energy efficiency. A double Q-learning algorithm that handles the problem of trajectory design of UAVs was presented in Reference [128]. Although standard Q-learning algorithms use the same Q-table for selection and evaluation processes and usually tend to overestimation and suboptimal results, this algorithm uses two Q-tables to decouple the selection from the evaluation. The effectiveness of the proposed algorithm was demonstrated via extensive simulations, and the gain was up to 19.4% and 6.7% regarding the number of satisfied users compared to the random algorithm and Q-learning algorithm, respectively.

Further advancements on UAV navigation were given in Reference [129]. In greater detail, a UAV navigation scheme based on DRL that can select the best UAV-to-ground links in real-time was presented for a massive MIMO system which included one ground station with a large number of antennas and multiple UAVs with single antennas. First, a DQN was constructed to extract the environment information and to obtain useful features of the massive MIMO channel, and then, the DQN was trained in order to facilitate the decision-making procedure based on the received signal strengths. Using the DQN, a Q-learning policy was also exploited for successfully optimizing UAV navigation while achieving increased coverage and rapid convergence. The high performance of the proposed DQN navigation scheme was confirmed through extensive simulation results, where the channel was Rician with a Rician factor of 6 dB and a ground station with 128 transmit antennas and 32 single-antennan UAVs flying at different velocities were considered.

Finally, the optimization problem of proper dynamic placement of UAVs as BSs in downlink scenarios was studied in Reference [130]. It was considered that the UAVs should make decisions about their placement in each time-slot, depending on the unknown density of ground users in an ML manner. Moreover, a constraint regarding the minimum UAV-recall-frequency or otherwise the maximum life-time, indicating the energy efficiency of mobile UAVs networks, should be also satisfied. In each time-slot, the UAV-recall-frequency was considered static and the results showed that the optimal UAV placement is achieved when the transmit power becomes equal to the onboard circuit power. In addition, the optimal hovering altitude that minimizes transmit power is proportional to the coverage radius, whereas the slope depends on the propagation environment and tends to increase in areas with high-rise buildings. These results also underlined that limiting on-board circuit power prolongs the life-time of mobile UAV networks. Next, the multiple time-slot scenario was studied, where unstable and non-ergodic time-varying density of ground users served with fixed data rate exists. For this scenario, the optimization problem was more complex and required a multi-stage decision process leading to an integer nonlinear programming coupled with an inherent integer linear programming. Since this problem was NP-hard, a sequential-Markov-greedy-decision (SMGD) method was proposed in order to achieve near-minimal UAV-recall-frequency in polynomial time. According to the results, a large number of sample sets are indispensable for effective pattern formation and reduction of UAV-recall-frequency in large areas with high-rise buildings and low ground user density whereas the SMGD becomes more complex as the number of UAVs increases.

Table 6 includes the relevant works on ML-based resource allocation and network planning techniques for UAV-enabled communication networks.

## 6. Open Issues

The AI/ML-empowered and learning-driven wireless networks will bring unique decision-making capabilities and real-time predictions towards revolutionizing 5G and beyond 5G networks. In this direction, the latest advancements in the AI/ML techniques have opened up new opportunities for the UAV-based systems and have led to the possibility of realizing highly autonomous UAV operations while enhancing the performance, safeguarding the security, and mitigating human faults under complex and dynamic scenarios. Nevertheless, there exist open research issues that require attention and should be addressed. The range of future research directions can be summarized as follows.

### 6.1. Practical Implementation

The AI/ML techniques rely, in principle, on massive and high-quality labeled data sets to achieve the desired results. Although the data availability is currently possible and economically and technologically less expensive due to the development of IoT sensor systems and cloud computing, the data collected by sensors and network equipment is usually subject to losses, redundancy, mislabeling, and class imbalance. Therefore, the effectiveness of the training procedure is questionable. Currently, the TensorFlow Lite, an open-source, framework developed by Google for ML inference on low-power embedded devices, can be used in order to succesfully exploit ML and to facilitate object recognition on unclassified data [131]. Moreover, Keras [132], a high-level neural networks application programming interface (API) written in Python, is capable of running on top of TensorFlow and enables fast experimentation. Moreover, today’s powerful multi-core central processing unit (CPU) architectures, GPUs, and broad availability of libraries for DL (e.g., [133]) allow for fast, parallel data processing in real-time. However, the limited battery capacity and on-board processing capabilities and power resources of the UAVs severely restrict the application of DL-based techiques, which allow for object detection, depth prediction, target tracking and localization, and decision-making on the fly. In particular, practical constraints mainly regarding the computational power, parallel data processing in real-time, and power consumption restrict the design and implementation of efficient DL solutions on UAVs. These issues are expected to be solved in the near future, as powerful miniaturized computing devices with low-power consumption are an active working field for embedded hardware developers. Hence, future research efforts should be devoted to further investigating and verifying the performance of AI/ML-aided aerial networks, especially in terms of the computation efficiency and hardware design. In this direction, future developments may also include the improvement of the processing time of the learning algorithms by adding a confidence score to predictions and a scale factor to the generated actions, which will lead to the reduction of the number of the required actions. Also, one could combine different ML techniques in order to cooperatively carry out the prediction procedure and thus to improve computational efficiency. Apart from including a larger number of samples for optimization, it could be also interesting to derive the optimal parameters of the learning algorithms to achieve faster convergence. For the special case of UAV swarms consisting of micro-drones with limited capabilities, the DL algorithms could be run on a BS with extensive computational power that will act as a central manager connected to the UAV mesh network. This base station will rely on the sensor data from all of the UAVs to determine the best possible action. Nevertheless, this control solution is not the ideal choice, since it often leads to increased signaling overhead and transmission latency owing to the necessary information exchange between the BS and the UAVs.

### 6.2. Physical Layer Issues

Currently, there is a gap in acquiring data from extensive measurement campaigns. As a future work, one could could implement test-beds carry out real experiments in different propagation areas, especially in dense, urban, skyscraper-rich settings and over sea areas [46] in order to validate the accuracy of the learning algorithms, especially in scenarios with dynamically changing environments and ground nodes moving at high velocities, e.g., vehicles, with increased latency and resiliency requirements, while considering the interference in the propagation area and inspecting at real-world constraints, such as the energy efficiency of the learned trajectory. Nevertheless, these real-world problems typically comprise high-dimensional continuous state spaces, i.e, large number of states and/or actions, and make the corresponding problems practically intractable with current approaches. As more measured data is acquired, ML-based approaches can foster new developments in UAV channel modeling. Also, ML methods can be applied beyond PL prediction, such as the power-delay profile, correlation coefficient and matrices, as well as collinearity among others [47]. Furthermore, interference mitigation poses a major hurdle towards the efficient integration of UAVs in future networks. ML could improve the performance of multiple techniques that have been considered in ground networks, such as power control, UAV-user association, and seamless handover using ML solutions for predicting user mobility and network load, while the use of ML in forming the precoding matrices of massive MIMO-enabled UAVs can eliminate the interference and can increase the quality of the transmission [134]. Another area that can be enhanced by ML is the clustering of users and UAVs towards improved NOMA in the downlink and uplink, increasing the chances of successful interference cancellation maximizing the spectral efficiency of the network [135]. Recently, the authors in Reference [136] investigated the joint optimization of throughput provided by UAV BS and energy that they sell and buy for recharging from the gird. Therefore, in scenarios where multiple UAVs are deployed to serve as aerial BSs, the joint consideration of physical-layer metrics and energy and the application of ML techniques, such as DL to process heterogeneous data, can offer increased performance, as network lifetime prolongation is a critical aspect of UAV networks.

### 6.3. Data Link and Network Layers Issues

In hierarchical based networks, several challenges exist when applying ML and DL techniques in MAC and routing protocols. For example, various parameters should be considered during the protocol design phase, such as the computation efficiency, the accuracy requirements, the acquisition of the communication parameters from all the network elements, the centralized or distributed control performance, and the architecture design. Distributed UAV swarm networks in varying formations and without centralized control should be also studied in order to handle inter-swarm communication optimization issues. To coordinate the data transmission in multi-UAV cooperative scenarios, ML methods that enable the grouping the UAVs into several clusters based on their location, type of sensors, and type of data are indispensable. In network planning and user association problems, energy efficiency for the UAV-aided networks should also be considered. Specifically, additional constraints in the optimization strategy could be included, such as the flight time and the total energy consumption, in order to obtain a trade-off between mobility and radio coverage and to maximize the amount of ground nodes and the flight time of the UAVs while satisfying the QoE. Moreover, in cluster-based UAV networks, resource management may be performed in a centralized or distributed manner. The first approach has a high computational cost, while cooperative decisions are very challenging, since the information should be shared among all the UAVs. The adoption of a suitable solution depends on various parameters, including the network architecture, system requirements, and the wireless environmental conditions. Proactive caching by UAVs is also a research field that can greatly benefit from ML as the wealth of available data related to user-centric information and network conditions can improve the placement of content, leveraging on the dynamic UAV deployment [88]. Regarding UAV scheduling, in Reference [137], a mathematical framework for the optimization of UAV-aided video monitoring of a set of points of interest (PoI) distributed in a large urban area was proposed. Using this framework, which is based on Mixed Integer Linear Programming (MILP) techniques and real experimental data, particular energy-constrained UAVs are selected for recharging using public transportation buses, which also transfer the UAVs to desired PoIs in order to increase reliability and coverage. Still, ML-based solutions relying on DNNs can enable MILP-based optimization to reach its full potential, as suggested in Reference [138].

### 6.4. Security and Privacy Issues

There is a strong need for advanced methods in combating ever evolving attack types against the reliability of communication [31] and the safety of critical infrastructures. Security concerns during the operation of UAV-based delivery systems include CP attacks targeted at hijacking the delivery UAV and damaging or stealing its load. Also, for multimedia systems relying on UAVs, disrupting the UAV’s transmissions by forging their identities might have a severe impact especially in networks comprising a large number of UAVs. In such cases, UAV authentication and analysis of the received multimedia content by the BS is required, resulting in excessive delay. Then, in intelligent transportation systems, swarms of UAVs cooperate and exchange data to support specific tasks. Nonetheless, these swarms are vulnerable to attackers that joint the swarm and inject false data that might result in errors during UAV movement and possible damage due to collisions. To cope with the risk of adversarial ML during raw data transmission, federated learning is suggested, in which the training data of a specific ML task is stored in a distributed fashion across the UAVs in the swarm and the optimization problem is handled collectively [139]. In ML-enhanced PLS scenarios, there are still some challenges that are not tackled by current solutions. In Reference [48], the Q-learning-based algorithm adapts the transmission according to the attack type of the malicious UAV. However, in each time, knowledge is required to reconfigure the transmission parameters. In addition, the anti-jamming PHC-based algorithm of Reference [50] can be further developed to provide an anti-jamming mechanism for VANETs at the initial phase of the learning process, while achieving low-complexity RL solutions are necessary to improve PLS performance against smart jammers. Next, in GPS-spoofing scenarios [52], online learning can further improve the security provided by the NN, while unsupervised ML, capable of handling unlabeled data, can perform classification prior to processing, reducing the delay and increasing the accuracy. To further improve the performance of current ML-inspired detection methods, future work could be devoted to combining these methods with other conventional detection techniques, such as camera images and videos, radar echoes, and acoustic recordings, and to exploiting the advantages of each method. Moreover, future work may also focus on using different types of UAVs with varying velocities for the same experiments, on investigating the effect of interference and noise, and on collecting measured data with a greater variety of controllers.

## 7. Conclusions

The integration of unmanned aerial vehicles in wireless communication networks has provided several possibilities for novel network paradigms with increased flexibility and performance. At the same time, the application of machine-learning techniques in dense wireless networks has allowed the development of low-complexity solutions for an overall network optimization from the physical layer up to resource and network management. In this survey, an extensive overview of the application of machine learning in networks with unmanned aerial vehicles has been presented. Moreover, a classification of these techniques based on the communication and network aspects that they are implemented in has been given. Also, open issues in each relevant area have been discussed, outlining the importance of radical improvements in wireless networks and the necessity for further advancements based on the application of machine learning in unmanned aerial vehicles communication networks. Overall, it is safe to say that, when vast amounts of data from multiple sources are available, deep learning-based solutions can satisfactorily reveal useful correlations among such heterogeneous data towards optimizing UAV networks. Still, in scenarios where UAVs have limited processing capabilities or where dedicated cloud/fog/edge infrastructure does not exist to process large amounts of data, while connectivity with and between the UAVs is not ensured, this approach might not be practically feasible. Therefore, considering the varying processing capabilities of UAVs, their energy constraints, and the need for autonomous and distributed coordination, solutions entailing lower complexity and local estimation and processing of parameters might be more appropriate. This is the reason why machine-learning solutions, e.g., reinforcement and regret-based learning, have been adopted in several relevant studies.

## Figures and Tables

**Table 1:** Relevant surveys and tutorials on artificial intelligence (AI)/machine learning (ML) and unmanned aerial vehicle (UAV) communications.

Reference	Short Description	Scope of AI/ML on UAV
Li et al. [4]	A survey on cyber-physical applications in multi-UAVs	ML for object detection and image recognition
Mozaffari et al. [5]	UAV cellular communications	ML for trajectory and placement
Fotouhi et al. [6]	Regulatory aspects of using UAVs for cellular communications	UAV trajectory and placement
Carrio et al. [22]	A review of DL for UAVs	DL for feature extraction, planning, situational awareness, and motion control
Luong et al. [23]	DRL in wireless networks	UAV as an agent
Zhang et al. [24]	A tutorial on UAVs for wireless networks	Trajectory and path planning
Qian et al. [25]	Wireless big data	No discussion on AI and ML
Saad et al. [26]	6G vision	No concern about AI and UAV
Tong et al. [27]	A survey of 5G and beyond for communication of UAV	Nothing specific
Ullah et al. [28]	A survey of V2X communications	Security in VANET with UAVs
Wang et al. [29]	A survey on UAV networks from the viewpoint of CP security	ML and RL at the physical layer and routing correspondingly
Shakeri et al. [30]	A comprehensive tutorial of ANN-based ML for wireless networks	RL for coverage, connectivity, trajectory, and path planning
Challita et al. [31]	Wireless and security challenges for different UAV use cases	ANN-based solutions for interference management
Alsamhi et al. [32]	AI techniques for communication between robots	Intelligent swarm robot communication
Kato et al. [33]	AI on SAGIN	Based on routing on a case study of a complete network
Chen et al. [34]	An overview of 5G communications for V2X, drones, and healthcare	No discussion on ML and UAV
This survey	A survey on AI/ML for UAV-based communications	AI/ML integration in UAV-based communications and open issues

**Table 2:** Communication issues in ML-enhanced UAV networks.

Reference	Communication Target	ML Solution
Goudos et al. [36,37]	UAV RSS prediction	ANN with DE LM training
Wang et al. [38]	Air-to-ground channel modeling	Unsupervised online
Chen et al. [39]	Radio map construction	Segmented regression
Egi et al. [40]	PL prediction in irregular terrains	ANN with LiDAR
Zhang et al. [41]	Air-to-air PL prediction (RF)	RandF and KNN
Yang et al. [42]	Air-to-ground PL prediction (mmWave)	RandF and KNN
Chen et al. [43]	Efficient opportunistic UAV channel access	Distributed log-linear
Challita et al. [44]	Air-to-ground interference mitigation	ESN-based RL
Athukoralage et al. [45]	Interference management among LTE-U and Wi-Fi networks	Regret-based
Zhang et al. [46]	Optimized AMC	DL with CNN and LSTM
Ren et al. [47]	Robust and energy-efficient precoding	CE-based

**Table 3:** Security issues in ML-enhanced UAV networks.

Reference	Security Target	ML Solution
Li et al. [51]	Eavesdropping mitigation	Q-learning
Xiao et al. [52]	Interference and jamming mitigation	PHC-based learning
Xiao et al. [53]	Jamming, spoofing, and eavesdropping mitigation	RL
Manesh et al. [54]	GPS spoofing protection	ANN-supervised learning
Hoang et al. [55]	Eavesdropping detection	One-class SVM and K-means
Min et al. [56]	Interception of malicious UAVs	Q-learning
Zohdi [35]	Real-time mapping	Genetic algorithm-based
Shoufan et al. [57]	UAV pilot identification	Classification based on LD, QD, SVM, KNN, or RandF
Liao et al. [58]	Guaranteeing privacy, integrity, and confidentiality of UAV compressed video streams	CNN-based detection
Yue et al. [59]	Protection against trespassing UAVs	SVM-based ML
Santiago et al. [60]	Small boat detection	DL-based CNN

**Table 4:** Resource allocation and network planning issues in ML-enhanced UAV networks.

Reference	Resource Allocation Target	ML Solution
Park et al. [61]	UAV connectivity prediction	LR and SVM
Zhang et al. [62]	Predict best serving cell	Conditional random field
Cheng et al. [63]	Connectivity recovery in disastrous area	k-means
Li et al. [64]	Reducing handoffs	RL
Zhang et al. [65]	Deploy UAVs BSs	Gaussian mixture model
Chen et al. [66]	Resource allocation	Echo state networks
Hu et al. [67]	Multi-modal multi-task offloading	Recurrent neural network
Yang et al. [68]	Dynamic task scheduling	RL
Shamsoshoara et al. [69]	Optimizing the PU and UAV network resources	Distributed RL
Anicho et al. [70]	HAPS network coordination	RL
Chen et al. [75]	UAV deployment and caching	ESN-based
Chen et al. [76,77]	UAV spectrum allocation and caching	LSM-based
Dai et al. [78]	UAV spectrum allocation and caching	RL

**Table 5:** Detection and localization issues in ML-enhanced UAV networks.

Reference	Detection and Localization Target	ML Solution
Alipour et al. [88]	Identification of UAV type over encrypted Wi-Fi traffic	MLE and l1-norm regularization
Amorim et al. [89]	AU detection from measured RF data	Bayesian, SVM, and MLP
Zhang et al. [92]	UAV classification from measured RF data	ANN
Ezuma et al. [93]	UAV classification from raw RF signal fingerprints	Markov-based naive Bayes detection, KNN, DA, SVM and NN-based classification
Matson et al. [94]	Detection of UAV flying path	SVM and CNN
Sciancalepore et al. [96]	Detection of UAV flying status	J48, RandF, and NN
Shorten et al. [100]	UAV controller detection from transmitted control signals	CNN
Mozaffari et al. [101]	Detection of UAV-UEs from sparse information	Kernel density estimation
Al-Sa’ad et al. [102]	Detection of UAV type and flight mode from raw RF signals	DNN
Opromolla et al. [104]	UAV detection in a swarm	DL

**Table 6:** Placement and trajectory issues in ML-enhanced UAV networks.

Reference	Placement and Trajectory Target	ML Solution
Liu et al. [109]	Throughput	Q-learning
Ladosz et al. [110]	Throughput	GP and NMPC based
Bayerlein et al. [111]	Sum-rate	Q-learning
Ladosz et al. [112]	Communication quality	NN
Liu et al. [113]	MOS and QoE	Q-learning
Peng et al. [115]	Mobility prediction and object profiling	Unsupervised learning
Esrafilian et al. [116]	Throughput and path planning	Map compression based
Colonnese et al. [118]	QoE	Q-learning
Dai et al. [119]	Sum-rate	Distributed learning
Jailton et al. [120]	Throughput	ANN
Klaine et al. [121]	Radio coverage	Q-learning
Ghanavi et al. [122]	QoS	Q-learning
Mozaffari et al. [101]	Latency	Kernel density estimation
Wu et al. [123]	Spectral efficiency	DQN
Hu et al. [124]	Coordination of multiple UAVs	Q-learning
Liu et al. [126]	Radio coverage	Decentralized DRL
Liu et al. [128]	QoS	Double Q-learning
Huang et al. [129]	Radio Coverage	DRL
Lu et al. [130]	Energy efficiency	SMGD

## Abbreviations

The following abbreviations are used in this manuscript:

3GPP	3rd Generation Partnership Project
AIS	Automatic Identification System
AMC	Adaptive Modulation and Coding
ANN	Artificial Neural Network
AP	Access Point
AU	Aerial User
BS	Base Station
CA	Collision Avoidance
CE	Cross Entropy
CEO	Cross Entropy Optimiser
CNN	Convolution Neural Network
CP	Cyber-Physical
CPS	Cyber-Physical System
CSI	Channel State Information
CSMA	Carrier Sensing Multiple Access
D2D	Device-to-Device
DA	Discriminant Analysis
DDPG	Deep Deterministic Policy Gradient
DDRL	Deep Distributed Reinforcement Learning
DQN	Deep Q-Network
DQL	Deep Q-Learning
DL	Deep Learning
DRL	Distributed Reinforcement Learning
eNB	Evolved NodeB
ESN	Echo State Network
FANET	Flying Ad Hoc Network
GEO	Geostationary Earth Orbit
GMM	Gaussian Mixture Model
GP	Gaussian Process
GPS	Global Positioning System
GPU	Graphics Processing Unit
GU	Ground User
HAPS	High Amplitude Platform Station
HDMF	Heterogeneous Deep Model Fusion
IoT	Internet of Things
KNN	K Nearest Neighbor
LAP	Low Altitude Platform
LD	Linear Discriminant
LEO	Low Earth Orbit
LiDAR	Light Detection and Ranging
LR	Linear Regression
LSBoost	Least Squares Boosting
LSM	Liquid State Machine
LTE	Long-Term Evolution
MAB	Multi-Armed Bandit
MAC	Medium Access Control
MAE	Mean Absolute Error
MAPE	Mean Absolute Percent Error
MDP	Markov Decision Process
MEO	Medium Earth Orbit
MFCC	Mel-Frequency Cepstral Coefficients
MG	Minority Games
MILP	Mixed Integer Linear Programming
MIMO	Multiple-Input Multiple-Output
ML	Machine Learning
MLE	Maximum Likelihood Estimation
MLP	Multiple Layer Perceptron
mmWave	millimeter Waveband
MOS	Mean Opinion Score
MUM	Manned and Unmanned
NASA	National Aeronautics and Space Administration
NHPSO	Network-Based Heterogeneous Particle Swarm Optimization
NMPC	Nonlinear Model Predictive Control
NN	Neural Network
NOMA	Non-Orthogonal Multiple Access
NTN	Non-Terrestrial Networks
OBU	On-Board Unit
OFDM	Orthogonal Frequency Division Multiplexing
OFDMA	Orthogonal Frequency Division Multiple Access
PHC	Policy Hill Climbing
PL	Path-Loss
PLS	Physical Layer Security
PoI	Point of Interest
POMDP	Partially Observable Markov Decision Process
PSN	Public Safety Network
PT	Prospect Theory
QD	Quadratic Discriminant
QoE	Quality of Experience
QoS	Quality of Service
RandF	Random Forest
RAT	Radio Access Technology
RF	Radio Frequency
RL	Reinforcement learning
RMSE	Root Mean Squared Error
RNN	Recurrent Neural Network
RPV	Remotely Piloted Vehicle
RSS	Received Signal Strength
RSU	Roadside Unit
RTCA	Radio Technical Commission for Aeronautics
SDR	Software Defined Radio
SINR	Signal-to-Interference plus Noise Ratio
SIR	Signal-to-Interference Ratio
SMGD	Sequential-Markov-Greedy-Decision
SNR	Signal-to-Noise Ratio
SSA	Salp Swarm Algorithm
SSN	Self-Sustaining Networks
STFT	Short-Time Fourier Transform
SVM	Support Vector Machine
TDMA	Time Division Multiple Access
UAV	Unmanned Aerial Vehicle
UE	User Equipment
UI	User Interface
USRP	Universal Software Radio Peripheral
V2X	Vehicle-to-Everything
VANET	Vehicular Ad Hoc Network
WPC	Wireless Powered Communication
